# The origin and evolution of Earth's nitrogen

**DOI:** 10.1093/nsr/nwae201

**Published:** 2024-06-12

**Authors:** Yuan Li

**Affiliations:** State Key Laboratory of Isotope Geochemistry, Guangzhou Institute of Geochemistry, Chinese Academy of Sciences, Guangzhou 510640, China; Bayerisches Geoinstitut, Universität Bayreuth, Bayreuth 95440, Germany

**Keywords:** nitrogen, isotopes, silicate Earth, atmosphere, magma ocean

## Abstract

Nitrogen is a vital element for life on Earth. Its cycling between the surface (atmosphere + crust) and the mantle has a profound influence on the atmosphere and climate. However, our understanding of the origin and evolution of Earth's nitrogen is still incomplete. This review presents an overview of the current understanding of Earth's nitrogen budget and the isotope composition of different reservoirs, laboratory constraints on deep nitrogen geochemistry, and our understanding of the origin of Earth's nitrogen and the deep nitrogen cycle through plate subduction and volcanism. The Earth may have acquired its nitrogen heterogeneously during the main accretion phase, initially from reduced, enstatite-chondrite-like impactors, and subsequently from increasingly oxidized impactors and minimal CI-chondrite-like materials. Like Earth's surface, the mantle and core are also significant nitrogen reservoirs. The nitrogen abundance and isotope composition of these three reservoirs may have been fundamentally established during the main accretion phase and have been insignificantly modified afterwards by the deep nitrogen cycle, although there is a net nitrogen ingassing into Earth's mantle in modern subduction zones. However, it is estimated that the early atmosphere of Earth may have contained ∼1.4 times the present-day atmospheric nitrogen (PAN), with ∼0.4 PAN being sequestered into the crust via biotic nitrogen fixation. In order to gain a better understanding of the origin and evolution of Earth's nitrogen, directions for future research are suggested.

## INTRODUCTION

Understanding the origin and evolution of Earth's nitrogen (N) would assist in the comprehension of atmosphere chemistry throughout Earth's geological history [1,2], the emergence and evolution of life on Earth [3], and the climate of the early Earth [4,5]. As a life-essential element, nitrogen also holds crucial clues for the possible presence of life on extraterrestrial planets [6]. Consequently, nitrogen geochemistry, encompassing its concentration, speciation, isotopes and cycling between Earth's atmosphere, crust and mantle, has been extensively studied since the pioneering work of Rayleigh (1938, 1939) [7,8], Stevenson *et al.* (1953) [9] and Haendel *et al.* (1986) [10]. Nevertheless, our understanding of the origin and long-term evolution of nitrogen in Earth's mantle and surface (atmosphere + crust) reservoirs remains incomplete [11–14].

Nitrogen constitutes 78% of the present-day atmosphere by volume. Nitrogen present as N_2_ in the atmosphere is chemically inert due to its strong triple bond; however, biotic processes can take it up and convey it into reactive species such as nitrite (NO_2_^−^), nitrate (NO_3_^−^) and ammonium (NH_4_^+^) in the biosphere [15–17]. All living organisms contain nitrogen, and the incorporation of nitrogen from organic matter into phyllosilicates via the substitution of NH_4_^+^ for K^+^ during diagenesis marks the entrance of nitrogen from the biosphere to the lithosphere [15,18,19]. This process results in the transition of nitrogen from behaving as a highly volatile element to a lithophile element. Nitrogen has two stable isotopes, ^14^N and ^15^N, and stable nitrogen isotope compositions are expressed as δ^15^N (‰) = [(^15^N/^14^N)_sample_/(^15^N/^14^N)_standard_–1)] × 1000, where the standard is the present atmospheric N_2_ with ^15^N/^14^N = 0.003676. The NH_4_^+^ of phyllosilicates would inherit the positive δ^15^N signature of organic nitrogen, which is why sedimentary and metasedimentary rocks usually have positive δ^15^N values [17,18,20]. During continuous metamorphism in the continental crust, accompanied by continuous devolatilization and loss of N_2_ and/or NH_3_, the δ^15^N of residual NH_4_^+^ in K-bearing minerals increases [10,21–24]. Dehydration melting of the continental crust can also lead to appreciable loss of nitrogen from the rock, depending on whether or not there are nitrogen host minerals in the melting residues [25]. Over the past 80 years, the concentrations and isotopes of nitrogen in crustal rocks (sedimentary, igneous and metamorphic rocks) have been extensively measured [8–10,13,18,24,26–32]; however, the data for the deep continental crustal rocks are relatively scarce [26].

Since the 1990s, mantle-derived rocks and gases (volcanic gases and hydrothermal fluids, mid-ocean-ridge basalts (MORBs), oceanic-island basalts (OIBs), diamonds, and mantle xenoliths) [33–45] have been studied in conjunction with subduction zone rocks [31,46–51]. The nitrogen concentration and isotope composition of these samples were measured thanks to the advancements of sophisticated analytical techniques that permit the analysis of sub-nanomole quantities of nitrogen in samples [52–56], such as CO_2_ laser extraction static gas mass spectrometry [54], continuous flow isotope ratio mass spectrometry [57] and modified noble gas mass spectrometry [53]. The new insights gained from these studies indicate that nitrogen can be incorporated into mantle minerals and rocks [43]. Furthermore, the mantle may constitute a significant portion of Earth's nitrogen inventory [13,58]. Additionally, the nitrogen isotope composition of the mantle differs from that of the surface, forming a long-standing unresolved puzzle referred to as ‘nitrogen isotope disequilibrium’ [12,59]. The difference in δ^15^N of Earth's mantle and surface makes nitrogen a useful tracer for the dynamic exchange of Earth's mantle and surface and the deep nitrogen cycle in Earth's history through plate subduction and volcanism [59]. The estimated present-day global nitrogen influx by plate subduction is overall larger than the outflux by volcanism, which indicates a net nitrogen ingassing into Earth's mantle [11,51,60]. However, the question remains as to whether such a net nitrogen ingassing can be applied to the ancient warm/hot subduction zones [4,51]. Consequently, the question of whether this net nitrogen ingassing indicates a higher N_2_ partial pressure of Earth's early atmosphere remains unanswered [4,11,51,61].

Over the past two decades, laboratory experiments have also been conducted with the objective of elucidating the geochemistry of deep nitrogen and the origin and evolution of Earth's nitrogen. The experiments were conducted at conditions that are relevant for the early Earth magma ocean, mantle melting, subduction zone processes and magmatic degassing within the shallow crust. In combination with high-precision *in situ* analyses using an electron microprobe [62,63], Fourier-transform infrared (FTIR) and Raman spectroscopy [64,65], secondary ion mass spectrometry (SIMS) [63,66–68], and NanoSIMS [64,69], these experiments reveal a significant redox dependence of nitrogen behavior and the transition of nitrogen from a highly volatile element to a lithophile or a siderophile element in geological materials; its solubility in mantle minerals and melts [67–74], its partitioning between minerals, fluids and silicate melts [61,75–77], and its partitioning and isotope fractionation between metallic and silicate melts in planetary magma oceans [78–83] all depend on redox conditions. These experimental results, when considered alongside observations of natural samples, provide preliminarily constraints on the accretion processes of Earth's nitrogen [78,81,84]. They also shed light on the distribution of nitrogen between the proto-Earth's core, mantle and surface [78,80,85,86], the storage of nitrogen in Earth's mantle [68,70,74,87], and the long-term evolution of nitrogen in Earth's mantle and surface after the core formation [61,77].

This review presents an overview of the observations on nitrogen abundances and isotope compositions of Earth's different reservoirs, and the estimated nitrogen influx and outflux in subduction zones. It also offers a general picture of the advancements of laboratory experiments performed thus far to understand the deep nitrogen geochemistry. The combined natural observations and experimental results are used to discuss the possible origin and long-term evolution of Earth's nitrogen. Finally, future research directions are proposed to address the remaining issues.

## EARTH'S NITROGEN BUDGET AND SPECIATION

### Nitrogen budget

**Figure 1.**
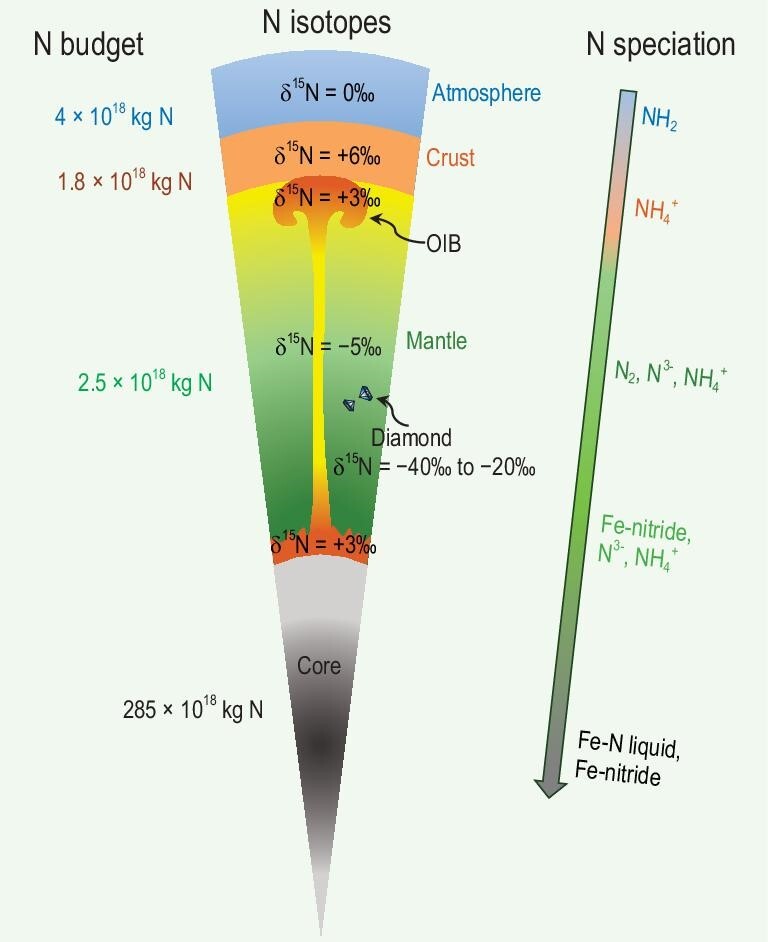
Nitrogen budget, isotope composition and speciation of Earth's different reservoirs. See the main text for the references and discussion. This figure is modified from Fig. 1 of Shi *et al.* (2022) [79] and is not to scale.

Figure 1 presents the current nitrogen budget of the Earth's surface, mantle and core reservoirs. The present-day atmosphere contains 4 × 10^18^ kg N_2_ [4], and the ocean nitrogen mass is 2.4 × 10^16^ kg with N_2_ as the dominant species and other minor species such as NO_3_^−^, NH_4_^+^ and N_2_O [58,88]. The nitrogen mass in biomass is relatively low, amounting to ∼9.6 × 10^14^ kg in total [88]. However, this reservoir plays a pivotal role in the transfer of nitrogen from the atmosphere into sediments and subsequently into the deep crust and mantle. This is achieved through biotic nitrogen fixation [19], which is an important process in understanding the deep nitrogen cycle on Earth. The nitrogen abundance in the crust can be estimated using the nitrogen content (typically in the range of a few μg/g to a thousand μg/g) of sedimentary, igneous and metamorphic rocks and their proportions [58]. The nitrogen content of the bulk continental crust has been estimated to be in the range of 50–88 μg/g [26,58,89]. Using the most recent value of 74 μg/g [26] yields a nitrogen mass of 1.4 × 10^18^ kg for the bulk continental crust. In the oceanic crust, the nitrogen stored in sediments has a mass of 0.32 × 10^18^ kg^4^, while the nitrogen stored in basaltic and gabbroic oceanic crust has a mass of 0.04–0.06 × 10^18^ kg [32]. Therefore, the total crustal nitrogen is ∼1.77 × 10^18^ kg.

The relatively constant N_2_/^40^Ar ratios observed in MORBs and the mantle ^40^Ar content derived from helium were used by Marty (2012) [14] and Marty and Dauphas (2003) [59] to estimate the nitrogen content of the MORB mantle source with an assumption that nitrogen and argon behave similarly during mantle melting and magmatic degassing. This was calculated to be 0.27 ± 0.16 μg/g nitrogen. Using a whole MORB mantle C/N ratio of 535 ± 224 and a mantle carbon content of 50 ± 25 μg/g [40], Marty (2012) derived a nitrogen content of 0.11 ± 0.07 μg/g in the whole MORB mantle (N- and E-MORBs). Using the N_2_/^36^Ar ratios of MORBs from Marty and Dauphas (2003) [59] and a mantle ^36^Ar content derived from helium, Bekaert *et al.* (2021) [11] estimated a range of 0.21–0.69 μg/g nitrogen for the MORB mantle, with a best estimate of 0.4 μg/g. For the plume mantle source, Marty and Dauphas (2003) [59] estimated a nitrogen content of 2.7 ± 1.4 μg/g using the N_2_–^36^Ar systematics of plume-related high ^3^He/^4^He samples. This value could be as high as 30 μg/g if the ^3^He-rich plume Yellowstone samples are used [90]. Using the limited variation in N_2_/^40^Ar ratios of MORBs and OIBs, and the estimated ^40^Ar budget of the bulk mantle, which represents the silicate Earth (excluding the atmosphere), Marty (2012) [14] estimated a nitrogen content of 0.84 ± 0.43 μg/g for the bulk mantle. Bergin *et al.* (2015) estimated a nitrogen content of 1.1 ± 0.55 μg/g for the convecting mantle, which is comparable to the high estimate values (0.08 ± 0.05 to 1.2 ± 0.5) of Halliday (2013) [91]. Marty (2012) [14] observed that the CI-chondrite-normalized nitrogen abundance in both the MORB mantle and the atmosphere is considerably lower than that of other volatiles, such as carbon and water. This was referred to as the ‘missing’ nitrogen. This also led to the formulation of the concept of the superchondrtic C/N ratio in the silicate Earth, namely that the silicate Earth exhibits a higher C/N ratio than the CI chondrites due to the greater depletion of nitrogen than carbon in the silicate Earth. Marty (2012) [14] proposed that a considerable quantity of nitrogen may have been incorporated into Earth's core during core formation. However, Johnson and Goldblatt (2015) [58] estimated a much higher nitrogen concentration (6 ± 4 μg/g) for the convecting mantle based on some mantle samples, which have N_2_/^40^Ar values up to two orders of magnitude higher than the N_2_/^40^Ar values of oceanic basalts. These two authors suggested that the plume mantle source could be rich in nitrogen. If the estimate of Johnson and Goldblatt (2015) [58] is reliable, it can remove the concept of missing nitrogen proposed by Marty (2012) [14]. However, the high N_2_/^40^Ar values of the mantle samples used by Johnson and Goldblatt (2015) [58] have been questioned because such high N_2_/^40^Ar values could be caused by contamination during step heating analyses [92].

If we use the most recently estimated MORB mantle nitrogen content of 0.4 μg/g [11] and the plume mantle nitrogen content of 2.7 μg/g [59], and if we employ the mass ratio of the MORB mantle vs. plume mantle of 9 : 1 [59], then we can calculate that the entire convecting mantle contains ∼2.52 × 10^18^ kg nitrogen. This figure, in conjunction with the crustal nitrogen mass, would be equivalent to the Earth's present-day atmospheric nitrogen mass. The total nitrogen mass of Earth's mantle, crust and atmosphere would then be ∼8.3 × 10^18^ kg (Fig. 1).

An analogy of Earth’s core may be the iron meteorites. Johnson and Goldblatt (2015) [58] estimated a core nitrogen mass of ∼250 × 10^18^ kg using a value of ∼140 μg/g nitrogen in iron meteorites and regarding iron meteorites as a proxy for Earth's core. If we use an average value of ∼24 μg/g derived from the iron meteorite data compiled in Grewal *et al.* (2021) [93], a core nitrogen mass of ∼43 × 10^18^ kg can be estimated. Partitioning of nitrogen between Earth's core and mantle (see below) was employed to estimate a nitrogen content of ∼160 μg/g for Earth's core [78], which would result in a core nitrogen mass of ∼285 × 10^18^ kg. These estimates indicate that the core may be Earth's largest nitrogen reservoir (Fig. 1).

### Nitrogen speciation

Nitrogen speciation undergoes changes in Earth's various reservoirs (Fig. 1). Crustal nitrogen is predominantly present as NH_4_^+^ in K-bearing minerals, which were inherited from organic matter as previously mentioned. The presence of NH_4_^+^ in amphibole of metasomatic origin was observed in mantle xenoliths [43], which serves to illustrate that NH_4_^+^ may also be stable in mantle rocks [43,94]. The presence of NH_4_^+^ in K-bearing minerals renders nitrogen a lithophile element, exhibiting similar behavior to K and Rb [43,47,95]. Nitrogen was also found in fluid inclusions in minerals from the crust and upper mantle, and Raman spectroscopy analyses indicate that the dominant nitrogen species is N_2_, without any detectable NH_3_ [94,96–98]. In volcanic gases the predominant nitrogen species is N_2_; only very minor NH_3_ was found in a few volcanic gas samples [99]. Nitrogen species in other forms (e.g. NO_X_, HNO_3_) in volcanic gases have also been reported [100]. However, the formation of such oxidized nitrogen species likely involves reactions with atmospheric oxygen. Furthermore, nitrogen that is present as nitride and carbonitride has been identified in diamonds from the deep reduced mantle, and nitride could be a significant host of nitrogen in Earth's deep upper mantle and lower mantle [101].

### Experimental constraints on nitrogen storage in Earth's interior

**Figure 2.**
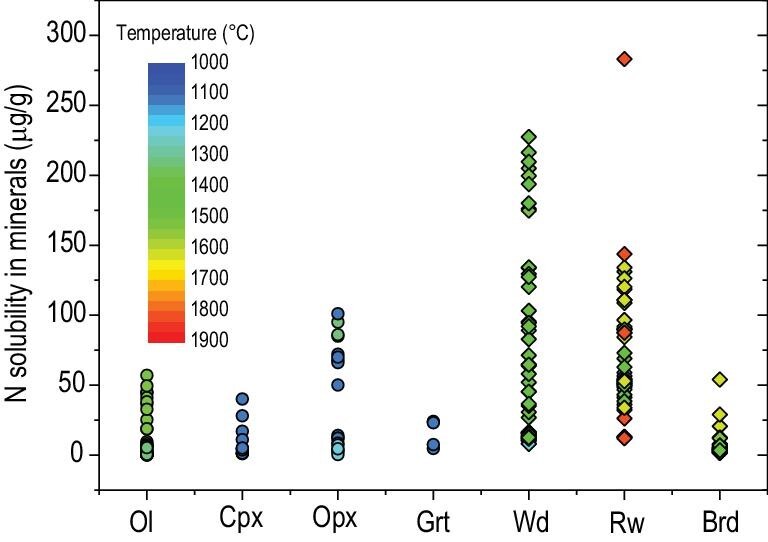
Nitrogen solubility in mantle minerals, obtained at conditions corresponding to the Fe–FeO buffer and the saturation of N_2_-rich gas. Nitrogen solubility generally increases with increasing temperature. The other parameters that potentially affect nitrogen solubility include mineral composition and pressure. For example, the presence of Al in Cpx and Opx can enhance nitrogen solubility. Ol = olivine; Cpx = clinopyroxene; Opx = orthopyroxene; Grt = garnet; Wd = wadsleyite; Rw = ringwoodite; Brd = bridgmanite. Data are compiled from references [68,70,74].

The determination of nitrogen solubility in mantle minerals at the saturation of N_2_-rich gas (Fig. 2) is of great importance for the understanding of the storage and distribution of nitrogen in Earth's mantle. Watenphul *et al.* (2010) synthesized NH_4_^+^-bearing diopside at 9.5–12.8 GPa and 725–750°C with nitrogen introduced as a 25% NH_3_·H_2_O solution [71]. The NH_4_^+^ concentration measured in diopside is up to 1000 μg/g. Li *et al.* (2013) [68] investigated the solubility of nitrogen in forsterite, diopside, enstatite and pyrope at *f*O_2_ buffered by Ni–NiO (NNO), Co–CoO (CoCoO) and Fe–FeO (IW), and at 1.5–3.5 GPa and 1000–1300°C. The nitrogen content of minerals buffered by NNO or CoCoO was at most a few μg/g at very high pressures. Very high nitrogen solubility up to 100 μg/g was observed at the IW buffer in enstatite at high temperatures or in Al-bearing enstatite and diopside. The nitrogen solubility in forsterite at the IW buffer also increased with temperature and pressure; a maximum solubility of 10 μg/g was obtained at 3.5 GPa and 1300°C. The strong enhancement of nitrogen solubility at reducing conditions may be related to nitrogen dissolution as either NH_4_^+^ or N^3−^, which directly substitutes for Na/K or O^2−^. The experimental results revealed that the reduced lower part of the upper mantle has the capacity to store ∼20–50 times more nitrogen than the present-day atmosphere. Yoshioka *et al.* (2018) [70] measured nitrogen solubility in mantle transition zone minerals wadsleyite and ringwoodite. The observed nitrogen solubilities in wadsleyite and ringwoodite typically ranged from 10–250 μg/g, with a strong increase in solubility with temperature. Their measured nitrogen solubility in bridgmanite was ∼30 μg/g. Fukuyama *et al.* (2023) [74] experimentally determined 2–6 μg/g nitrogen in bridgmanite, which also increased with increasing temperature. More recently, it was shown that wüstite and Fe–N may form a solid solution in Earth's deep mantle [87]. All these experimental results demonstrate a significant nitrogen storage capacity of the Earth's mantle, indicating that nitrogen may not be anomalously depleted in the silicate Earth but reside in a reservoir in the deep Earth that is poorly sampled.

The data on nitrogen in metal are useful for understanding the capacity of Earth's core and deep reduced mantle to store nitrogen. Experiments performed in diamond anvil cells [102] demonstrated that Fe–N compounds can be stable at extremely high temperatures and pressures corresponding to the conditions of Earth's core. Other experiments also demonstrated the stability of titanium nitride, iron nitride and carbonitride from deep upper mantle to core pressure [103–105]. These results indicate that nitrogen is siderophile at high *P*–*T* and reducing conditions, that nitrogen can be stored as nitride in Earth's deep mantle, and that nitrogen is a potential light element in Earth's core (Fig. 1). Early studies of nitrogen solubility in liquid metals at the saturation of N_2_-rich gas were typically related to steel production. It was found that nitrogen solubility in liquid metal obeys Sievert's law [106], which states that nitrogen solubility in liquid metal is proportional to $\surd {{P}_{{{N}_2}}}$, where ${{P}_{{{N}_2}}}$is the partial pressure of N_2_. Recent experimental studies, which were more relevant to the Earth's core and mantle [80,83,107,108], showed that nitrogen solubility in Fe-rich liquid metal, as high as 12 wt%, increased strongly with increasing pressure but decreased moderately with increasing Ni content at *P*–*T* conditions up to 18 GPa and 2580°C. The presence of sulfur, carbon and/or silicon may also decrease the nitrogen solubility in Fe-rich liquid metal [80,109]. Overall, these experimental results reveal that core-forming liquid metal can dissolve large quantities of nitrogen, and that Earth's core could be a large nitrogen reservoir. Furthermore, nitrogen in the deep reduced mantle may be mainly stored in the small metal fractions due to the large metal–silicate nitrogen partition coefficients [108].

## EARTH'S NITROGEN ISOTOPE COMPOSITION

### Nitrogen isotopes

The δ^15^N values of the present-day upper mantle, as inferred from fibrous diamonds and MORBs, range from −10‰ to 0‰. These values converge towards a globally uniform value of −5‰ [33,59], which has been widely accepted as the depleted mantle value [12,110]. The overall positive δ^15^N values of the organic matter and metasediments indicate that the crustal material is enriched in ^15^N compared to the atmosphere [12,15]. The δ^15^N value of Earth's surface is approximately +3‰ [12], and a δ^15^N value of +4.3‰ was estimated recently for the bulk continental crust [26], although some crustal rocks such as the Altay granites have negative δ^15^N values (down to −7‰) [111]. These isotope data indicate that the majority of crustal nitrogen is of biotic origin, although a notable fraction may be of mantle-derived, magmatic origin [39]. Slab altered oceanic crust (AOC) and serpentinized mantle peridotite [12,30–32,60] and most metamorphic rocks from subduction zones [21,47–49,51,112] have overall positive δ^15^N values, although negative δ^15^N values also exist, for example, some blueschists and eclogites show δ^15^N values as low as −16‰ due to abiotic nitrogen reduction [113,114]. Deep mantle materials sampled by mantle plumes (plume lavas and OIBs) have been found to have δ^15^N values ranging from −2‰ to +8‰, with a mean value of approximately +3‰ [37,59]. Using the rare ^15^N^15^N isotopologue of N_2_ as a novel tracer of air contamination in volcanic gas effusions, Labidi *et al.* (2020) [90] found that the Eifel (Germany) and Yellowstone (USA) gases exhibited mantle-derived δ^15^N values of −1.4 and +3‰, respectively. Notably, these values are also higher than the mantle value of −5‰. However, diamonds derived from the mantle transition zone and lower mantle exhibited similar δ^15^N values to the upper mantle, with the exception of a few diamonds exhibiting the lowest δ^15^N values (ranging from −25‰ to −40‰) [115]. The positive δ^15^N values of plume lavas and OIBs have been interpreted as being the result of the addition of recycled sediments to the mantle source region [37,59]. Nevertheless, the majority of diamond populations with an Archean age exhibited a narrow range of δ^15^N values, spanning −12‰ to +5‰, with a mode around −5‰ [44]. This indicates that there has been no significant secular change in mantle δ^15^N, suggesting that nitrogen recycling has been limited. Using the largest available data set of δ^15^N in mantle diamonds, Stachel *et al.* (2022) [116] recently showed that mantle diamonds exhibit a large range of δ^15^N values, spanning −40‰ to +17‰. The observed mode value was found to be −3.5‰, which is slightly higher than the accepted mantle value. The reason for the formation of such a large range of δ^15^N could be due to nitrogen isotope fractionation and fluid mixing; however, the authors demonstrated that the mantle sources for nitrogen in diamonds may not have undergone a significant isotopic change between ∼3.5 and 0.7 Ga, which also points to limited nitrogen recycling. Accordingly, the negative δ^15^N value of −5‰ observed in the Earth's mantle may have been established prior to the Archean period, and the positive δ^15^N values observed in mantle plume-related materials may represent primordial signatures of the deep mantle [90,110].

The extremely negative δ^15^N values, down to −25‰ and −40‰, found in a few diamonds were also interpreted to be relicts of primordial nitrogen and used to argue for an enstatite chondrite (EC) origin of Earth's nitrogen [12,115,117]. This was because the δ^15^N values (ranging from −45‰ to −15‰) of ECs [118] are more closely aligned with those observed for Earth's mantle rather than those of carbonaceous chondrites (δ^15^N = +15‰ to +55‰) [119]. If the nitrogen on Earth was derived primarily from EC-like materials, it is necessary to understand how the nitrogen on Earth evolved from the initial δ^15^N values of −45‰ to −15‰ to the mantle value of −5‰ and the positive δ^15^N value of Earth's surface. Additionally, it is necessary to re-evaluate whether the positive δ^15^N values of mantle plume-related materials are of primordial origin or a secondary origin.

### Nitrogen isotope disequilibrium between Earth's mantle and surface

The discrepancy in δ^15^N between Earth's mantle (−5‰) and surface (+3‰) cannot be explained by mantle degassing and was therefore referred to as the nitrogen isotope disequilibrium [12]. If Earth's surface nitrogen were derived from mantle degassing, one would expect negative δ^15^N values of Earth's surface, because light nitrogen ^14^N is preferentially lost during degassing [120]. Several models have been proposed to account for the nitrogen isotope disequilibrium. (i) The heterogeneous accretion model [117] suggested that the Earth's accretion history was composed of two parts. The first part was dominated by EC-like materials with δ^15^N values as low as −40‰, while the second was a ‘late veneer’ with highly positive δ^15^N values (ranging from +30‰ to +40‰) that may have consisted of CI-chondrite-like materials and a minor cometary component. The subsequent mantle degassing and subduction would have decreased the surface δ^15^N values but increased the mantle δ^15^N values. The extremely negative δ^15^N values down to −25‰ and −40‰ found in a few mantle diamonds were the relicts of primordial nitrogen in the mantle, which could have been isolated from mantle convection and homogenization. This model would result in ∼30 μg/g nitrogen in Earth's mantle, which is significantly higher than any previously mentioned estimates for the Earth. Furthermore, a CI-chondrite-like veneer was questioned recently, based on ruthenium isotopes [121,122]. (ii) The hydrodynamic model [123] assumed that the Earth's accretion materials had negative nitrogen isotope compositions similar to ECs (−30‰), as proposed by Javoy (1997) [117]. Nitrogen with negative δ^15^N values was incorporated into the primitive mantle, and a portion of the mantle nitrogen was subsequently released into the atmosphere due to impacts and/or magma-ocean–atmosphere interactions. The primitive atmospheric ^14^N was then preferentially lost into space due to the aerodynamic drag of light gases such as hydrogen and helium, resulting in an increase of δ^15^N to approximately +2.5‰ relative to the modern atmosphere. Following the closure of the atmosphere to nitrogen loss, the δ^15^N decreased towards its present-day value due to mantle degassing. However, a hydrodynamic nitrogen loss from the early atmosphere is inconsistent with Earth's isotope compositions of noble gases and other major volatiles [14]. (iii) The core–mantle interaction model [36,37] assumed that the segregation of liquid Fe–Ni alloys into the core may have preferentially extracted ^14^N and left a ^15^N-enriched mantle. Some experimental results [79,124] support nitrogen isotope fractionation between Earth's core and mantle. This model may explain the δ^15^N difference between Earth's mantle and the enstatite chondrites, but it does not explain the nitrogen isotope difference between Earth's mantle and surface. (iv) The nitrogen recycling model [4,59] postulated that the nitrogen in Earth's mantle originated from recycled atmospheric and crustal nitrogen. The upper mantle δ^15^N may correspond to the subduction of sediments in the Achaean, which had negative δ^15^N values, while the nitrogen in the mantle plumes may correspond to the subduction of sediments after the great oxidation event (GOE), which had positive δ^15^N values. Nevertheless, some of the Archean sediments also exhibited positive δ^15^N values, which were employed to challenge this model [125]. If the recycled materials in ancient subduction zones were enriched in ^15^N and the nitrogen degassed at ancient mid-ocean ridges had negative δ^15^N values, as observed in modern Earth, over time the mantle δ^15^N and the atmosphere δ^15^N should have evolved towards positive and negative values, respectively. Therefore, the nitrogen recycling model cannot resolve the nitrogen isotope disequilibrium.

## ORIGIN OF EARTH'S NITROGEN AND THE DISTRIBUTION OF NITROGEN ON THE EARLY EARTH

### Models for the accretion of Earth's nitrogen

Both planetary dynamic models [126,127] and geochemical evidence [128,129] indicate the delivery of volatiles by volatile-rich asteroids to the inner solar system. However, the mechanism and timing for the accretion of Earth's major volatiles, including nitrogen, remain a controversial topic [14,91,130,131]. Some authors have proposed that the Earth accreted its volatiles from CI-chondrite-like materials in the form of an undifferentiated ‘late veneer’ after core-formation ceased [132–134], similar to the model proposed by Javoy (1997) [117]. However, other groups have argued that the Earth accreted its volatiles from oxidized chondritic materials at its full or late accretion stages. In this model, the volatiles participated in the core-formation process at Earth's magma ocean conditions [127,135–137]. Additionally, some models proposed that the Earth acquired its volatiles from a single giant impactor, such as the Moon-forming impactor [81,138,139].

The δ^15^N of the proto-solar nebula was as low as −380‰, based on solar wind ions sampled by the Genesis mission [140]. The δ^15^N values of comets, obtained from spectroscopic observations of the CN, HCN and NH_2_ of comae, are as high as +1000‰ [141,142]. In light of the Earth's mantle δ^15^N of −5‰, simple mixing of the proto-solar nebular and cometary nitrogen can apparently explain the mantle δ^15^N; however, neither the proto-solar nebula nor the comets was suggested to be a substantial source of Earth's nitrogen based on combined nitrogen, hydrogen and noble gas isotopes [129,142,143]. The CI- and CM-chondrite-like materials have an average δ^15^N of +42‰ to +175‰ [129]. These δ^15^N values demonstrate that CI- and CM-chondrite-like materials cannot be considered the sole source of nitrogen on Earth. An EC origin of Earth's nitrogen is essentially consistent with the observation that the silicate Earth and ECs have largely identical isotopic compositions for O, Ca, Ti, Cr, Ni, Mo and Ru [121,122]. However, if EC-like materials are Earth's main nitrogen source, the δ^15^N of Earth's surface remains to be explained. The addition of CI-chondritic late veneer to the proto-Earth [117] may have caused an increase in Earth's mantle δ^15^N to −5‰. However, the mantle Ru-isotopes may rule out an outer solar system origin of the late veneer [121,122]. The non-chondritic relative volatile abundance in the bulk silicate Earth [14,91] also argues against the late veneer as an important source of Earth's major volatiles. Therefore, it can be postulated that Earth's nitrogen may have been delivered mainly during the main accretion phase [143]. Piani *et al.* (2020) [144] demonstrated that the hydrogen and nitrogen isotope composition of Earth's surface and mantle can be explained by the combination of EC and CI-chondrite-like materials. However, in this case, the EC and CI-chondrite-like materials should not have been delivered simultaneously. It is more probable that the CI-chondrite-like materials were delivered later, as the Earth's surface has higher δ^15^N and δD values than the mantle [144].

### Experimental constraints on nitrogen geochemistry in Earth's magma ocean

The inner-solar-system planets, including Earth, were formed largely from the accretion of numerous planetesimals and Moon- to Mars-sized embryos, which had already differentiated into a metallic core and silicate mantle [136,145]. Consequently, the quantity and distribution of volatiles in such differentiated bodies must have played a significant role in shaping Earth's volatile budget and ratios, if Earth's volatiles were delivered during the main accretion phase [81,84,138,146,147]. The fate of volatiles in a planetary magma ocean is determined by their dissolution and partitioning among the core, mantle and atmosphere [81,130,145,148]. Therefore, the solubility and partitioning of nitrogen in Fe-rich metallic and silicate melts at conditions relevant for magma oceans of planetesimals and embryos have been investigated with the objective of elucidating the origin of nitrogen and the superchondritic C/N ratio observed in the bulk silicate Earth.

**Figure 3.**
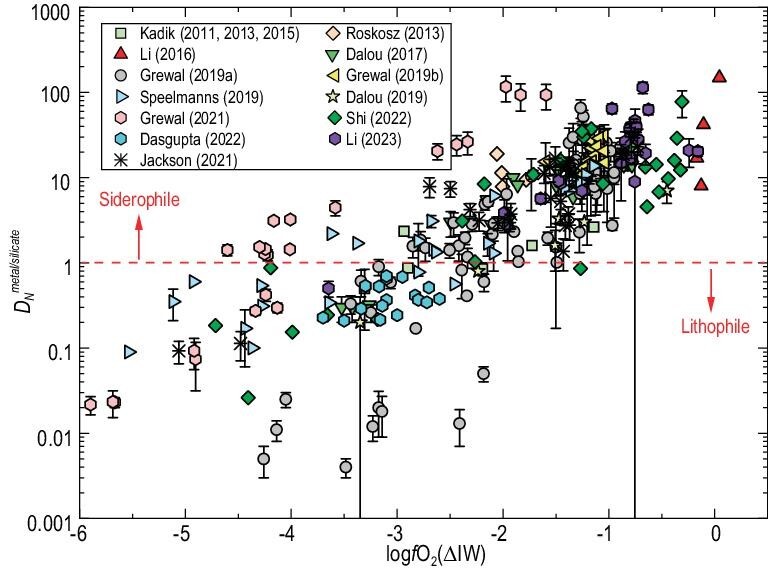
Metal–silicate melt partition coefficient of nitrogen ($D_N^{{metal}/silicate}$) as a function of oxygen fugacity relative to the Fe–FeO buffer (log*f*O_2_ (ΔIW)). Oxygen fugacity plays a major role in affecting $D_N^{{metal}/silicate}$, and the scattering of $D_N^{{metal}/silicate}$ can be ascribed to the variation in *P*–*T*, silicate melt composition and metal composition. A parameterization of $D_N^{{metal}/silicate}$ as a multifunction of these parameters is given in Shi *et al.* (2022) [78]. Data are compiled from references [78–81,83,84,107,124,149,150,158,159,190].

The metal–silicate melt nitrogen partition coefficient ($D_N^{{metal}/silicate}$) was determined at 1–26 GPa, 1673–3437 K and *f*O_2_ of IW-7 to IW, which are ∼0.01–100 and are strongly controlled by *f*O_2_ (Fig. 3) [78,80,82–86,107,149]. The nitrogen solubility in silicate melts at the saturation of N_2_-rich gas ($S_N^{{silicate}}$) and at *f*O_2_ < IW was determined at pressures up to 3 GPa (see below), and also shows a strong dependence on *f*O_2_ [67,72,83,150]. The *f*O_2_-dependence of $D_N^{{metal}/silicate}$ and $S_N^{{silicate}}$ is primarily attributable to the transformation of nitrogen speciation in silicate melt from N_2_ dominance at oxidizing conditions to N–H and N^3−^ dominance at reducing conditions. Previous studies have also determined $D_C^{{metal}/silicate}$, which are ∼10–10^5^ and largely higher than $D_N^{{metal}/silicate}$ (see a most recent study [83] for a review). The metal/silicate nitrogen isotope fractionation was determined at 1–8 GPa and 1400–2200°C. Shi *et al.* (2022) [78] demonstrated that metal–silicate melt nitrogen isotope fractionation factors, ranging from −4‰ to +10‰, are significantly influenced by *f*O_2_, probably also due to the change of nitrogen speciation in silicate melts. Kinetic processes may cause much larger metal–silicate melt nitrogen isotope fractionations, reaching up to 300‰ [79]. Nevertheless, more recent experimental studies and *ab initio* calculations [151] showed that metal–silicate melt nitrogen isotope fractionation is limited (ranging from –3.3‰ to –1.0‰) at 2–3 GPa and 1400–1700°C.

Using their $D_N^{{metal}/silicate}$ and $D_C^{{metal}/silicate}$, Grewal *et al.* (2019, 2022) [81,84] proposed that a single large impactor or undifferentiated planetesimals delivered Earth's nitrogen. Li *et al.* (2023) [83] demonstrated that nitrogen-to-carbon fractionation could occur during core formation and silicate magma ocean degassing. For a rocky body that begins with a chondritic C/N ratio, core formation would result in a superchondritic C/N ratio in its core if that rocky body is S- and Si-poor; however, a superchondritic C/N ratio can also be achieved in the silicate mantle through C-saturation coupled with preferential nitrogen degassing and loss into space, if the rocky body has a S-rich or Si-rich core. Both the accretion of planetesimals and embryos with cores as the major nitrogen and carbon reservoirs, and the disequilibrium accretion of C-saturated embryos through core–core merging, could have established the superchondritic C/N ratio in the bulk silicate Earth. Furthermore, the authors proposed that during Earth's accretion of the last few giant impactors, multiple episodes of magma ocean degassing and erosion-induced atmospheric loss would have also favored the formation of superchondritic C/N ratios in the bulk silicate Earth. This was due to the oxidized nature of Earth's surface magma ocean (*f*O_2_ > IW) and the preferential nitrogen degassing and loss into space. However, these studies do not consider nitrogen isotopes.

**Figure 4.**
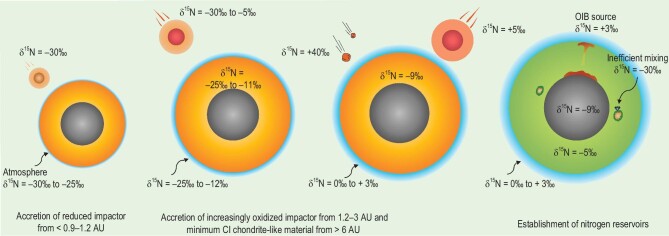
Earth's continuous but heterogeneous accretion of nitrogen during the main accretion phase. The Earth first obtained nitrogen from enstatite chondrite-like, reduced impactors from the inner solar system (<0.9–1.2 AU), then from increasingly oxidized impactors from 1.2–3 AU, and from minimal CI-chondrite-like oxidized materials. The nitrogen isotopes, as well as nitrogen budget, of Earth's mantle and surface reservoirs can be explained by these accretion processes. Note that the diamonds with largely negative δ^15^N (−30‰) may record the nitrogen isotope signature of early accreted reduced materials, and the OIB mantle source with positive δ^15^N may record the nitrogen isotope signature of late accreted oxidized materials. This figure is modified from Fig. 4 of Shi *et al.* (2022) [78]. See the main text for detailed discussion.

Shi *et al.* (2022) [78] applied their nitrogen isotope fractionation data and partitioning data to a multistage core-formation model [152], which was further refined by combining it with Grand Tack N-body accretion simulations [136]. They considered that the Earth accreted the first 60% of its mass through the collisions of reduced, EC-like impactors, and the last 40% of its mass through the collisions of increasingly oxidized impactors (Fig. 4). Reduced, EC-like impactors were formed at heliocentric distances of <0.9–1.2 AU with δ^15^N = −30‰, while increasingly oxidized impactors originated from greater heliocentric distances (1.2–3 AU). After the Earth accreted 60% of its total mass, a small quantity of completely oxidized or CI-chondrite-like materials, which contained ∼1000 μg/g nitrogen with δ^15^N = +40‰ and were formed from beyond 6–7 AU, was delivered to the Earth's magma ocean [153]. In addition, the authors considered other factors that potentially affected the nitrogen content and isotope composition of Earth's different reservoirs. These included the formation of a proto-atmosphere, the equilibrium degree between the silicate magma ocean and the proto-atmosphere, the surface magma ocean *f*O_2_, and the catastrophic loss of the proto-atmosphere during each collisional accretion. Their model results can explain the presently observed nitrogen content and δ^15^N of Earth's mantle and surface reservoirs and the superchondritic C/N ratio in the silicate Earth. Furthermore, the results revealed that Earth's core may contain ∼160 μg/g nitrogen with δ^15^N close to −9‰, which accounts for >90% of Earth's bulk nitrogen. The results of Shi *et al.* (2022) [78] showed that the surface nitrogen budget after Earth's core formation was ∼1.6 times the present-day atmospheric nitrogen mass and its δ^15^N was approximately +2‰. The authors thus suggested that Earth's nitrogen content and δ^15^N were the natural outcome of Earth's complex accretion processes. Chen and Jacobson (2022) [147] and Gu *et al.* (2024) [146] have also combined experimental $D_N^{{metal}/silicate}$, $D_C^{{metal}/silicate}$ and N-body accretion simulations in order to gain further insight into the processes that have shaped the volatile compositions of the Earth. The authors reached similar conclusions regarding the superchondritic C/N ratio in the silicate Earth, which they attributed to a complex interplay between the delivery of volatile-bearing planetesimals/embryos, core–mantle partitioning, mantle degassing and impact-induced atmospheric loss.

The results of Shi *et al.* (2022) [78] suggest that Earth's main nitrogen reservoirs and their nitrogen isotope compositions, including the nitrogen isotope disequilibrium between Earth's mantle and surface, may have already been established during the main accretion phase, and the subsequent evolution after Earth's formation through volcanism and plate subduction may have had a negligible effect. The success of the Shi *et al.* (2022) [78] model in explaining the nitrogen isotope disequilibrium between Earth's mantle and surface was contingent upon the heterogeneous accretion of reduced (inner solar system origin) to oxidized (outer solar system origin) materials, which exhibited largely negative and positive δ^15^N values, respectively. Due to its late accretion, the oxidized materials contributed less nitrogen to the mantle but more nitrogen to the surface. In contrast, the reduced materials contributed more nitrogen to the mantle but less nitrogen to the surface. Therefore, the heterogeneous accretion could explain the nitrogen isotope disequilibrium. Shi *et al.* (2022) [78] also suggested that the largely negative δ^15^N features of some deep mantle diamonds (Fig. 1) could be primordial, representing the relicts of inhomogeneous mantle mixing of EC-like impactors, as suggested previously [12,115,117], and that the positive δ^15^N features of plume mantle source could also be primordial, representing a long-term preservation of materials from oxidized impactors, such as those from the Moon-forming giant impactor (Fig. 4).

## THE DEEP NITROGEN CYCLE

**Figure 5.**
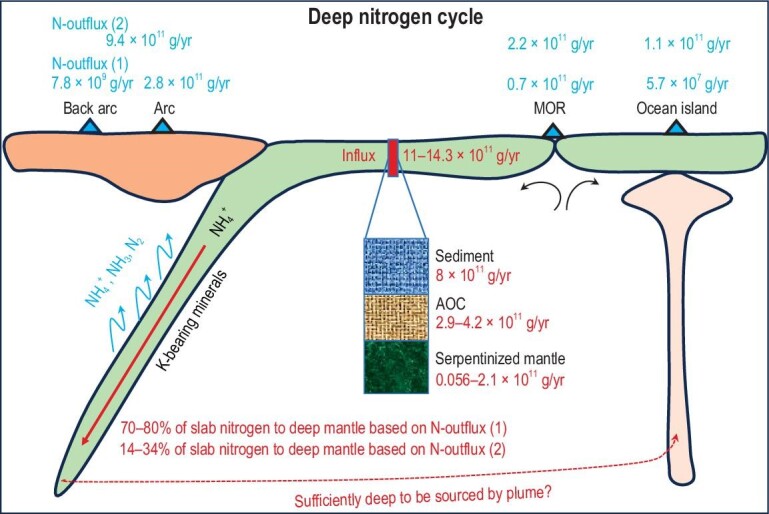
The deep nitrogen cycle. Nitrogen is present as NH_4_^+^ in K-bearing minerals in the slab. The nitrogen influx of sediment, altered oceanic crust (AOC) and serpentinized mantle is taken from a previous compilation [60]. The nitrogen outflux (1) is taken from references [34,41,51], and the nitrogen outflux (2) taken from Bekaert *et al.* (2021) [11]. During slab dehydration, nitrogen in the form of NH_4_^+^, NH_3_ and N_2_ is released into the sub-arc mantle wedge and then extracted by arc magmas. About 70%–80% of slab nitrogen is subducted into the deep mantle based on nitrogen outflux (1), and 14%–34% subducted into the deep mantle based on nitrogen outflux (2). Whether slab nitrogen can be subducted to the plume mantle source is debated [90,176].

The deep nitrogen cycle on Earth is of fundamental importance for the assessment of the distribution of nitrogen between the Earth's surface and mantle. The flux of nitrogen between these two major reservoirs may have undergone fluctuations throughout geological time, which have in turn influenced the evolution and composition of Earth's atmosphere. The main pathway for the release of mantle nitrogen into Earth's surface is through volcanism, whereas subduction zones represent the most significant locations for the return of surface nitrogen to the mantle (Fig. 5).

### Mantle nitrogen degassing

Partial melting of the depleted MORB mantle, sub-arc mantle and plume mantle has been the most important mechanism for mantle degassing since the Archean era [11]. The estimated nitrogen abundance in the depleted MORB mantle is ∼0.4 μg/g [11,14], and the predominant nitrogen species (N_2_) is only a very minor component of the erupted volcanic gases [99]. These observations imply that the nitrogen outflux from Earth's mantle may not have contributed significantly to the total nitrogen budget of Earth's present-day atmosphere. However, the present-day total mantle nitrogen outflux estimated by Busigny *et al.* (2011) [51] is ∼3.6 × 10^11^ g/yr. This figure was derived by combining the nitrogen outflux from mid-ocean ridges (0.7 × 10^11^ g/yr) [51], arc volcanism (2.8 × 10^11^ g/yr) [41] and intraplate volcanism (5.7 × 10^7^ g/yr) and back-arc basins (7.8 × 10^9^ g/yr) [34]. These nitrogen outflux values were calculated from N/^3^He, N/^36^Ar or C/N ratios of MORBs and arc volcanic gases and hydrothermal fluids, and these values may be subject to a ±50% uncertainty [34,51]. Bekaert *et al.* (2021) [11] estimated the present-day total mantle nitrogen outflux through the combination of nitrogen outflux from arcs (9.4 × 10^11^ g/yr), the depleted MORB mantle (2.2 × 10^11^ g/yr) and the plume mantle (1.1 × 10^11^ g/yr), which yielded 12.7 × 10^11^ g/yr. The authors employed the N/S ratio and sulfur outflux to compute nitrogen outflux from arcs, and they used an assumed constant degree of partial melting, partition coefficients, their estimated mantle nitrogen abundances, and magma production rates to compute the nitrogen outflux from the depleted MORB mantle and plume mantle. The nitrogen outflux estimated by Bekaert *et al.* (2021) [11] is significantly higher than that estimated by Busigny *et al.* (2011) [51], although Bekaert *et al.* (2021) acknowledged that their estimate represents an upper limit.

If we assume that the global nitrogen outflux has been constant over the past 3 billion years and apply the above-estimated nitrogen outflux data [11,51], the total mantle nitrogen outflux into the atmosphere would have represented 30%–90% of the present-day atmospheric nitrogen mass. If the nitrogen outflux has a mantle nitrogen isotope signature of −5‰, this would indicate that the early atmosphere must have had largely positive δ^15^N values, which are inconsistent with available observations on the early atmosphere δ^15^N (see below). This would suggest that the ancient mantle nitrogen outflux may have been smaller than the current estimates.

### Experimental constraints on nitrogen solubility and partitioning

**Figure 6.**
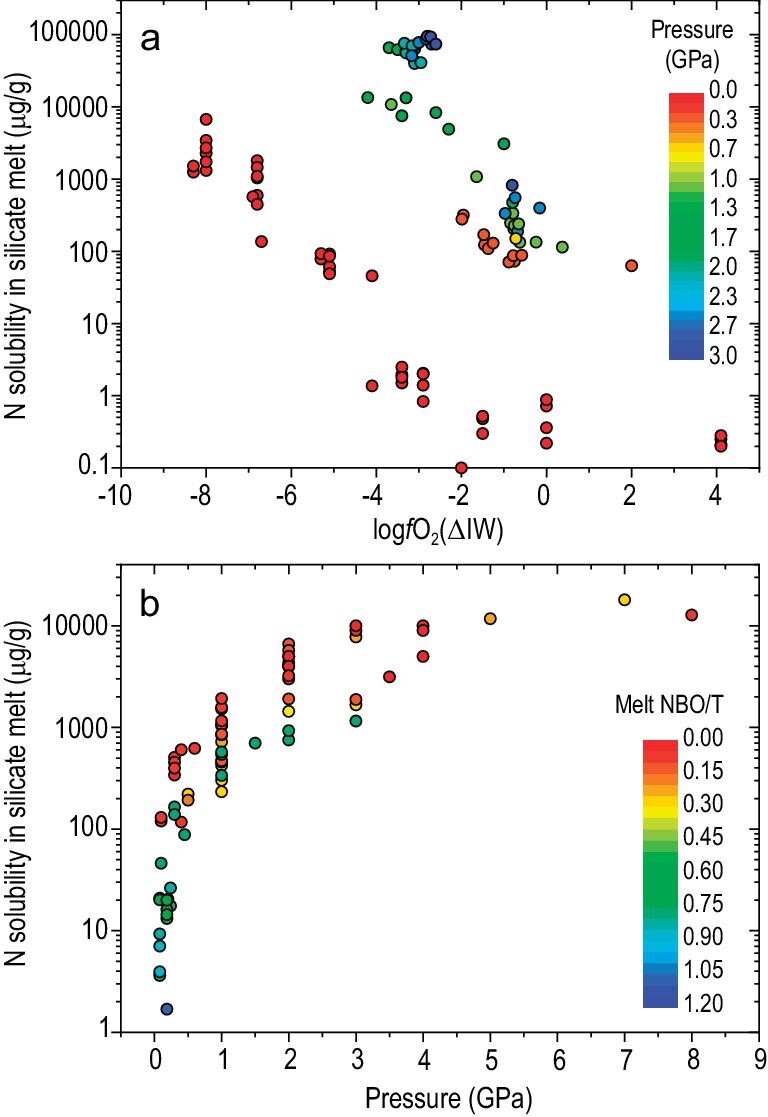
Nitrogen solubility in silicate melt at the saturation of N_2_-rich gas as a function of oxygen fugacity and pressure. (a) Nitrogen solubility increases with decreasing oxygen fugacity relative to the Fe–FeO buffer (log*f*O_2_ (ΔIW)) and increasing pressure. The nitrogen species in silicate melt changes from N_2_ dominance to N-H and N^3−^ dominance with decreasing oxygen fugacity. Compiled data are from references [67,72,83,150,191]. (b) Nitrogen solubility (physical dissolution as N_2_) in silicate melts at *f*O_2_ > IW increases with increasing pressure and decreasing silicate melt NBO/T (the ratio of non-bridging oxygens to tetrahedral cations). Compiled data are from references [64,73,154,160,184].

Knowledge of nitrogen solubility in silicate melts at the saturation of N_2_-rich gas ($S_N^{{melt}}$), nitrogen partitioning between mantle minerals and silicate melt, and nitrogen partitioning between fluid and silicate melt, is fundamentally important for understanding and quantifying the efficiency of mantle nitrogen degassing. Experimental studies focusing on determining $S_N^{{melt}}$ were conducted on dry basaltic/chondritic melts [67,72,107,154], hydrous/anhydrous silicate melts in the simplified SiO_2_ – Na_2_O ± FeO ± Al_2_O_3_ systems [155–159], and on hydrous basaltic and granitic melts at conditions relevant for partial melting of Earth's upper mantle and crust [61,64,73,160]. The most important results of these studies reveal that $S_N^{{melt}}$ depends on *f*O_2_, pressure and silicate melt composition (Fig. 6). The *f*O_2_-dependence of $S_N^{{melt}}$ reflects the changes of N-species dissolved in the silicate melts. At oxidizing conditions (*f*O_2_ > IW) nitrogen dissolves primarily as molecular N_2_ via physically filling the interstitial sites within the silicate melt network; however, at reducing conditions (*f*O_2_ < IW) nitrogen dissolves mainly as N–H and/or N^3−^ species through chemical bonding to the silicate network [61,62,65,67,72,154–156,158,161]. These findings indicate that the predominant nitrogen species in magmatic melts of Earth's upper mantle and crust is N_2_, as the *f*O_2_ values of most terrestrial magmas are above FMQ-2 [162]. Gao *et al.* (2022) [64] demonstrated that the $S_N^{{melt}}$ at *f*O_2_ > IW is smaller than the argon and CO_2_ solubilities at given conditions, indicating the fractionation of N_2_ from Ar and CO_2_ during the degassing of MORBs and arc magmas. Using all available nitrogen and argon solubility data obtained at *f*O_2_ > IW, Gao *et al.* (2022) [64] developed an empirical solubility model that can be used to predict the solubilities of nitrogen and argon in the silicate melts of Earth's mantle to crustal magmas. In contrast, Dasgupta *et al.* (2022) [150] developed an empirical model to predict nitrogen solubility in silicate melts at various oxygen fugacities. These models can be employed to elucidate the degassing of nitrogen in magmas of different redox conditions.

**Figure 7.**
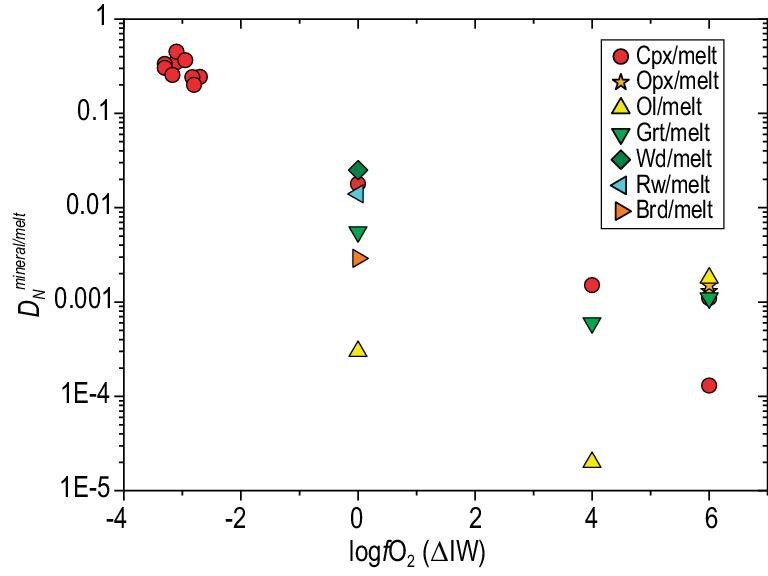
Mineral–silicate melt partition coefficient of nitrogen ($D_N^{{mineral}/melt}$) as a function of oxygen fugcity relative to the Fe–FeO buffer (log*f*O_2_ (ΔIW)). The data were compiled from references [68,70,150,160]. Note that the $D_N^{{mineral}/melt}$ of references [68,70] were estimated using nitrogen solubility in minerals and silicate melts. Ol = olivine; Cpx = clinopyroxene; Opx = orthopyroxene; Grt = garnet; Wd = wadsleyite; Rw = ringwoodite; Brd = bridgmanite.

Using their measured nitrogen solubility in mantle minerals and previously measured nitrogen solubility in silicate melts [156,158], Li *et al.* (2013) [68] calculated the partition coefficients of nitrogen between upper mantle minerals and silicate melts ($D_N^{{mineral}/melt}$) at various *f*O_2_ conditions. More recently, Dasgupta *et al.* (2022) [150] determined $D_N^{{mineral}/melt}$ for mantle clinopyroxene. All $D_N^{{mineral}/melt}$ values indicate that nitrogen is incompatible in mantle minerals with respect to silicate melts, but $D_N^{{mineral}/melt}$ increase with decreasing *f*O_2_ (Fig. 7). These results demonstrate that oxidizing conditions are more favorable for nitrogen degassing in the Earth's mantle. Li *et al.* (2015) [62] studied the nitrogen partitioning between aqueous fluids and silicate melts, with the starting melt composition corresponding to haplogranitic, basaltic and albitic melts at 0.1–1.5 GPa, 800–1200°C and *f*O_2_ ranging from IW to NNO + 3. The measured fluid/melt partition coefficients for nitrogen ($D_N^{{fluid}/melt}$) ranged from 60 for reduced haplogranitic melts to 10^4^ for oxidized basaltic melts. The most important parameters controlling $D_N^{{fluid}/melt}$ are *f*O_2_ and, to a lesser extent, melt composition. These partitioning data imply that nitrogen degassing from both MORBs and arc magmas is very efficient.

### Nitrogen subduction

The mass of nitrogen that is subducted into the mantle beyond the sub-arc depth can be estimated from the nitrogen influx at trenches (via slab sediments, AOC and serpentinized mantle peridotite) and by subtracting the nitrogen outflux of arcs (Fig. 5). The available estimates of the global slab nitrogen influx range from 13.2 × 10^11^ g/yr [51], 8.6 × 10^11^ g/yr [48,61], 10.5–11.8 × 10^11^ g/yr [32] and 15.4 × 10^11^ g/yr^11^, to 10.9–14.3 × 10^11^ g/yr [60]. The net slab nitrogen influx can be estimated in the range of 5.8–11.5 × 10^11^ g/yr, based on the global nitrogen outflux of 2.8 × 10^11^ g N/yr at arcs estimated by Hilton *et al.* (2002) [41]. This means that ∼20%–32% of the slab nitrogen is returned to the atmosphere, with the remainder primarily preserved in potassic minerals within the slab and subducted into the deep mantle. However, if we use the arc nitrogen outflux of 9.4 × 10^11^ g/yr estimated by Bekaert *et al.* (2021) [11], 62%–100% of the slab nitrogen would return to the atmosphere, and 66%–86% of the slab nitrogen would return to the atmosphere if we only consider the most recently estimated slab nitrogen influx by Li *et al.* (2023) [60]. Therefore, it is uncertain whether nitrogen can be massively cycled to the deep mantle beyond the sub-arc depth, due to the large discrepancy in the estimated nitrogen outflux at arcs [11,41].

Nitrogen is predominantly present in the form of NH_4_^+^ in K-bearing minerals, including feldspar, mica, amphibole, phlogopite and phengite, in slab sediments and AOC. Consequently, one crucial mechanism that determines whether nitrogen can be subducted into the deep mantle is the stability of K-bearing minerals in subduction zones [47,49,163–165]. In modern subduction zones, biotite can be stable at pressures up to 2.5–3 GPa [166], phengite up to 10 GPa [166,167], and hollandite beyond 12 GPa [168]. Therefore, phengite and hollandite may be the principal minerals responsible for the subduction of slab nitrogen into the deep mantle. However, during the dehydration or melting of the subducted slab, the actual amount of NH_4_^+^ retained in the K-bearing minerals would depend on the partitioning of nitrogen between the K-bearing minerals and the dehydrating fluids or hydrous melts [164], which is a multifunction of slab *P*–*T*–*f*O_2_ and fluid pH. Therefore, the deep subduction efficiency of slab nitrogen would strongly depend on the aforementioned parameters. A consensus has been reached among researchers studying subduction zone rocks that a significant portion of slab nitrogen (>60%) has been subducted into the deep mantle in cold subduction zones [48,49,51,60,112,169,170]. In addition, it has long been recognized that hot slabs have a higher devolatilization efficiency than cold slabs [95]. For instance, the hot slab of Central America (CA) may have lost its entirety of sedimentary nitrogen into the sub-arc mantle [171], whereas the cold slab of Izu-Bonin-Mariana (IBM) may have transported 90% of its sedimentary nitrogen and the majority of AOC nitrogen down to the mantle beyond the sub-arc depth [169]. However, a recent study by Li and Li (2022) [30] suggested that, based on the mass balance between the newly estimated nitrogen influx and outflux in the CA and IBM subduction zones, their deep nitrogen subduction efficiencies (55%–85% vs. 83%–88%) are not obviously different. It may be the case that the effect of slab thermal structure is only remarkable when the slab thermal structure extends to extremely hot subduction zones. If this is the case, then the global nitrogen outflux at arcs estimated by Bekaert *et al.* (2021) [11] may be an overestimation.

If we take the most recent estimation of the slab nitrogen influx by Li *et al.* (2023) [60], regardless of the nitrogen outflux at arcs estimated by Hilton *et al.* (2002) [41] or Bekaert *et al.* (2021) [11] being used, there is a net nitrogen influx into the deep mantle (Fig. 5). Since most of the slab metamorphic rocks have positive δ^15^N values, as mentioned previously, the positive δ^15^N values observed in some lamproites [172], some deep diamonds [173,174], and plume lavas and OIBs [59,175] were interpreted as evidence for the subduction of slab nitrogen into the deep mantle. However, the possibility that slab nitrogen can be subducted into the plume mantle source has recently been challenged, and it has been suggested that the nitrogen in the plume mantle source could be of primordial origin [90,176] (see also previous sections).

### Experimental constraints on nitrogen behavior in subduction zones

Laboratory experiments and thermodynamic simulations have been conducted to understand the geochemical behavior of nitrogen in subduction zones. Watenphul *et al.* (2009) [177] synthesized the NH_4_^+^- analogues of the K-bearing phases phengite, K-cymrite, K-Si-wadeite, K-cymrite and K-hollandite at 4–12.3 GPa and 700–800°C. These results suggest that high-pressure K-bearing minerals in subduction zones can store a significant quantity of NH_4_^+^ and possess the capacity to transport nitrogen into the deep mantle. The ratios of NH_4_^+^/N_2_ and NH_3_/N_2_ in subduction zone fluids are strongly influenced by *P*–*T*–pH–*f*O_2_; high pressure, low temperature and low *f*O_2_ result in elevated NH_4_^+^/N_2_ and NH_3_/N_2_ ratios [178–180]. Consequently, the deep nitrogen subduction efficiency depends not only on the thermal structure of the slab, but also the *f*O_2_. A slab that is hot and oxidized does not favor deep nitrogen subduction, whereas a cold and reduced slab would facilitate the recycling of nitrogen into the deep mantle.

**Figure 8.**
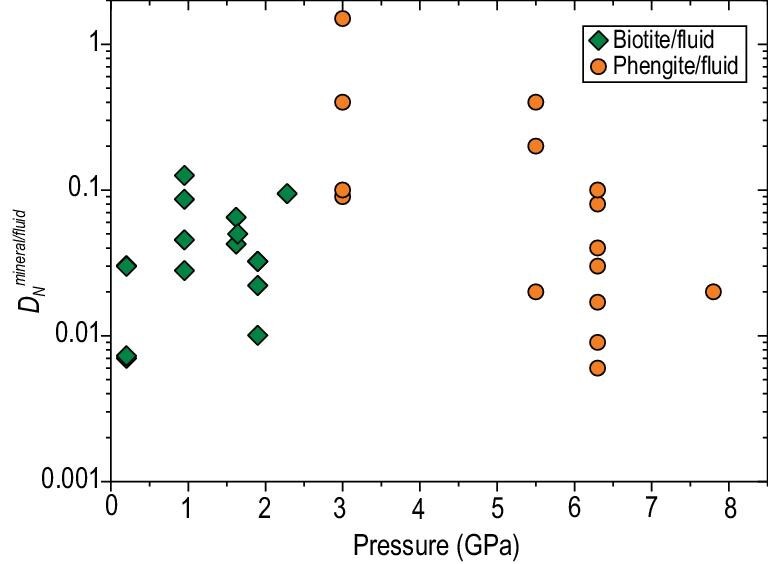
Mineral–fluid partition coefficients of nitrogen ($D_N^{{mineral}/fluid}$) as a function of pressure. The $D_N^{{mineral}/fluid}$ for biotite, taken from reference [182], generally increase with increasing pressure, while the $D_N^{{mineral}/fluid}$ for phengite, taken from references [75,77,192], show a decrease with increasing pressure. Note that the large scattering of $D_N^{{mineral}/fluid}$ at a given pressure is caused by the variation in oxygen fugacity and the concentration of nitrogen in fluid [75,76].

The $D_N^{{mineral}/fluid}$ and $D_N^{{mineral}/melt}$ have been determined for K-bearing minerals at 0.2–8 GPa and 400–1200°C and various *f*O_2_ (NNO − 5 to NNO + 2). Pöter *et al.* (2004) [181] measured $D_{NH_4^ + }^{{mineral}/fluid}$ for muscovite, tobelite, K-feldspar and phlogopite at 0.2–1.5 GPa and 400–600°C. At 1.5 GPa and 600°C, the $D_{NH_4^ + }^{{muscovite}/fluid}$ are ∼0.2. At 2–4 GPa, Föster *et al.* (2019) [77] obtained $D_N^{{phlogopite}/melt}$ of ∼0.8 and $D_N^{{phengite}/melt}$ of ∼0.9, and $D_N^{{fluid}/melt}$ of 0.4–3.7. Jackson *et al.* (2021) [182] measured $D_N^{{biotite}/fluid}$ of 0.007–0.2 and $D_N^{{biotite}/melt}$ of 0.1–3, and found that the variation in these values can be attributed to the effects of pressure and *f*O_2_. These data indicate that hydrous fluid is the preferred phase for nitrogen compared to biotite and silicate melt. The application of their partitioning data to slab dehydration *P*–*T* paths highlights the potential for highly incompatible behavior of nitrogen ($D_N^{{biotite}/fluid}$ < 0.1) from the slab along warmer and oxidized (NNO + 1) slab geotherms, whereas dehydration along reduced and cool geotherms will extract moderate amounts of nitrogen ($D_N^{{biotite}/fluid}$ > 0.1). However, Jackson and Cottrell (2023) [76] showed that both $D_N^{{biotite}/melt}$ and $D_N^{{melt}/fluid}$ increase as the nitrogen concentration in the system decreases within natural ranges, which implies that deep nitrogen subduction is more favored than the constraints proposed by Jackson *et al.* (2021) [182]. Kupriyanov *et al.* (2023) [180] and Sokol *et al.* (2023) [71] reported $D_N^{{phengite}/fluid}$ and $D_N^{{phengite}/melt}$ of 0.006–0.41 at pressures of 3–7.8 GPa and temperatures and *f*O_2_ common to hot oxidized slabs. The $D_N^{{phengite}/fluid}$ decrease sharply with increasing pressures (Fig. 8). This, in combination with the changeover of the nitrogen host from biotite to muscovite at the sub-arc depths and the subsequent decrease in phengite abundance in slab sediments, may lead to a significant slab nitrogen degassing. However, it is noteworthy that Sokol *et al.* (2023) [75] discovered the production of K-cymrite in the pelite system at pressure ≥6.3 GPa, and its bulk nitrogen content was 3.2–5.9 wt%, including 1.4–1.6 wt% NH_4_^+^, up to 0.5 wt% NH_3_ and 4–6 wt% N_2_. Thus, K-cymrite may act as a hidden redox-insensitive nitrogen reservoir in the mantle, involved in the deep nitrogen cycle. In conclusion, the inference from the results of laboratory experiments and thermodynamic calculations are in qualitative agreement with the natural observations that hot and oxidized slabs favor the release of slab nitrogen into the sub-arc mantle wedge, while cold and reduced slabs favor nitrogen subduction into the deep mantle beyond the sub-arc depth. Nevertheless, the experimental results have not yet been sufficient to permit a quantitative evaluation of the nitrogen recycling efficiency in subduction zones of different thermal structures. Furthermore, a recent study by Förster *et al.* (2024) [183], based on experimentally measured $D_N^{{mica}/fluid}$ of 0.1–0.2 at 2–2.7 GPa and 300–800°C, concluded that nitrogen partitions preferentially into fluid at high pressure and low temperature. The authors proposed that the recycling of nitrogen into the deep mantle is probably rare in modern subduction zones, whereas it is favored in hot subduction zones. This proposal appears to conflict with our previous understanding of nitrogen cycling in subduction zones.

## LONG-TERM EVOLUTION OF EARTH'S NITROGEN RESERVOIRS

A N_2_-rich atmosphere during the Archean could help resolve the ‘faint young sun’ paradox [4], which describes the apparent contradiction that, with the young Sun's output at only 70% of its current output, the early Earth would be expected to be completely frozen, but it seems to have had liquid water [5]. This paradox can be explained by pressure broadening of the absorption lines of greenhouse gases, which occurs when the atmosphere is N_2_-rich [4]. Goldblatt *et al.* (2009) [4] proposed that the nitrogen mass of the Archean atmosphere was about twice that of the present-day atmospheric nitrogen (PAN), with 1 PAN having been subducted into the deep mantle. This subduction origin of Earth's mantle nitrogen is consistent with the similar N/K ratios between Earth's surface and mantle [14,58]. A study based on nitrogen and noble gas isotope composition of the oceanic basalts from the Central Indian Ridge-Réunion plume region predicted a net nitrogen influx in subduction zones. The application of this net nitrogen influx as a constant through time yielded a 50% higher atmospheric density in the Archaean atmosphere than in the present-day atmosphere [177]. A high N_2_ partial pressure of the Archean atmosphere was also proposed by a few studies that applied the net nitrogen influx in modern subduction zones to the past [77,184]. These studies also used the present-day net nitrogen influx to calculate the total nitrogen mass subducted into Earth's deep mantle by assuming a constant nitrogen recycling efficiency in Earth's history. To explain the observed contrast in δ^15^N between Earth's surface and mantle, these studies assumed a high concentration of primordial nitrogen (∼6 μg/g) in Earth's mantle, with δ^15^N values (ranging from −6‰ to −7‰) only slightly lower than the present-day mantle δ^15^N value.

However, there is evidence that the nitrogen budget and isotope composition of Earth's surface and mantle have remained nearly constant throughout Earth's history. The estimates derived from fossilized rain-drop imprints in tuffs [185] and from vesicle size distribution in the Archean basalts [186] indicate that the N_2_ partial pressure of the Archean atmosphere may have been 0.5 bar 2.7 billion years ago. Marty *et al.* (2013) [187] analyzed fluid inclusions trapped in 3.0- to 3.5-billion-year-old hydrothermal quartz. Their results indicated that the N_2_ partial pressure of the Archean atmosphere was lower than 1.1 bar, potentially as low as 0.5 bar. Additionally, their results showed that Archean atmosphere exhibited a nitrogen isotope composition comparable to that of the present-day atmosphere. These studies indicate that the N_2_ partial pressure of the Archean atmosphere 2.7–3.5 billion years ago was not higher than that of the present-day atmosphere. The analyses of paleoatmosphere trapped in cherts indicated that the atmospheric δ^15^N of ∼0‰ has been constant over the past 3 billion years [188]. The studies of the δ^15^N of fluid inclusions preserved in 3.5 Ga hydrothermal deposits indicated that the δ^15^N of N_2_ dissolved in the 3.5 Ga seawater was −0.7‰ to −0.2‰, which are comparable to the present-day atmospheric value and also suggest a limited variation of the atmospheric δ^15^N in Earth's history [97]. Furthermore, as previously stated, mantle diamonds do not exhibit a secular change in mantle δ^15^N over 3 billion years [44,116]. These observations collectively indicate that there has been no significant exchange of nitrogen between Earth's surface and mantle.

A straightforward explanation for the near-constant δ^15^N of Earth's atmosphere and mantle over 3 billion years could be that the nitrogen budget and isotope composition of Earth's mantle and surface were formed during the main accretion phase and have been insignificantly modified by the subsequent deep nitrogen cycle through volcanism and plate subduction. This explanation is possible given that the total nitrogen mass released by MORBs (the present-day MORB nitrogen outflux is 0.7 × 10^11^ g/yr according to Busigny *et al.* (2011) [51]) may be very small compared to 1 PAN. Furthermore, the steep geothermal gradient of ancient slabs may have caused effective return of slab nitrogen back to the atmosphere through slab melting and/or dehydration. A significant quantity of nitrogen could have been subducted into the deep mantle only when slabs evolved to intermediate and cold *P*–*T* paths. If the nitrogen budget and isotope composition of Earth's surface and mantle were formed during the main accretion phase, then it should be noted that the nitrogen mass in the very early atmosphere before the presence of effective nitrogen fixation should have been ∼1.4 PAN (i.e. the total nitrogen mass of the present-day atmosphere and crust), given that a significant proportion of nitrogen in Earth's present-day crust is of biotic origin through atmospheric N_2_ fixation. The emergence of biotic nitrogen fixation should have significantly decreased the N_2_ partial pressure of the ancient atmosphere, accompanied by an increase in the nitrogen budget in the continental crust [189].

I perform some simple calculations below to demonstrate that the establishment of the nitrogen budget and isotope composition of Earth's mantle and surface during the main accretion phase is a more plausible explanation for the observed nitrogen budget and isotope composition than other potential models.

- **The mantle nitrogen was of pure surface origin.** If the mantle nitrogen with δ^15^N of −5‰ was of pure surface origin through slab subduction with negative δ^15^N before the GOE as suggested by Marty and Dauphas (2003) [59], then using a mantle nitrogen abundance of 0.4 μg/g [11], the total nitrogen subducted into the mantle would have to be 0.4 PAN before the GOE. This is highly implausible, given the elevated slab geothermal conditions before the GOE and the short duration of subduction history, if plate subduction commenced at ∼3 Ga [174].
- **The mantle nitrogen was, in part, of surface origin.** If half of the mantle nitrogen (0.4 μg/g) was of surface origin through slab subduction with a δ^15^N value of +4‰ after the GOE, then the δ^15^N of primordial nitrogen in the mantle would have to be −14‰, which is significantly lower than the nearly unchanged mode δ^15^N values of −3‰ to −5‰ recorded in mantle diamonds over 3 billion years [44,116]. In this model, a lower nitrogen abundance in Earth's mantle allows for more limited room for the addition of slab nitrogen. A high primordial nitrogen abundance (e.g. 1–5 μg/g) of the mantle would permit the addition of more surface nitrogen without significantly altering the mantle δ^15^N from −5‰, as demonstrated in Mallik *et al.* (2018) [61] and Förster *et al.* (2019) [77]. Nevertheless, the presence of a primordial nitrogen abundance of 1–5 μg/g in the Earth's mantle was not substantiated until the present day [11,14,91].
- **A high mantle nitrogen outflux but a low deep nitrogen subduction efficiency in Earth's history.** This model assumes that the Archean atmosphere had a lower N_2_ partial pressure, and the Archean mantle had a higher nitrogen abundance, compared to their present-day counterparts. If we use a nitrogen mass of 0.5 PAN for the Archean atmosphere, then the δ^15^N of the Archean atmosphere must have been +5‰ in order to obtain the present-day atmospheric δ^15^N of 0‰ through the degassing of MORB with a δ^15^N value of −5‰. This is inconsistent with the observed δ^15^N values for paleoatmosphere [97,187,188].
- **A high N_2_ partial pressure of the Archean atmosphere and a high nitrogen deep subduction efficiency in Earth's history.** If we assume that the nitrogen mass of the Archean atmosphere was 2 PAN and that 1 PAN has been subducted into the mantle [4] through intermediate to cold slabs with a positive δ^15^N, then the Archean mantle would have to be much more negative in δ^15^N, and the Archean atmosphere would have to be much more positive in δ^15^N, compared to their present-day counterparts. All available observations of the nitrogen budget and isotopes of Earth's mantle and surface are incompatible with this model.

## CONCLUSIONS AND FUTURE RESEARCH DIRECTIONS

As a volatile element, nitrogen present as N_2_ is highly concentrated in Earth's atmosphere. However, the Earth's core and mantle also represent significant nitrogen reserves, due to the storage of nitrogen as a lithophile element in the form of NH_4_^+^ and/or N^3−^ in silicates, and as a siderophile element in metals. The nitrogen isotope compositions of Earth's surface and mantle are different, resulting in a disequilibrium. Globally, there is a net nitrogen influx in modern subduction zones; however, the estimated fractions of the slab nitrogen that has been subducted into the mantle beyond the sub-arc depth have large variations due to the large uncertainty in the estimated nitrogen outflux at arcs. The non-secular change of the mantle and surface nitrogen isotopes and atmospheric N_2_ partial pressure would be consistent with a primordial origin of Earth's mantle nitrogen and a limited exchange of nitrogen between the mantle and the surface. Therefore, the deep nitrogen cycling may have not considerably modified the nitrogen budget and isotope composition of Earth's mantle and surface. The combination of planetary formation models and experimental constraints on the nitrogen partitioning and isotope fractionation between metallic and silicate melts in Earth's magma ocean reveals the establishment of the Earth's mantle and surface nitrogen reservoirs during the main accretion phase. The nitrogen mass of Earth's very early atmosphere before effective biotic nitrogen fixation could have been 1.4 PAN, equivalent to the PAN mass plus the crustal nitrogen mass.

However, despite decades of effort, our understanding of the origin and evolution of Earth's nitrogen remains incomplete. Further analysis of high-grade metamorphic rocks is necessary to better constrain the nitrogen budget of Earth's continental crust. It is possible that a significant proportion of nitrogen in the continental crust originated from the mantle, but the relative proportions of biotic and abiotic nitrogen remain unknown. The reduced lower upper mantle, mantle transition zone and the lower mantle all have a large nitrogen storage capacity; however, it remains to be tested whether a hidden nitrogen-rich reservoir, which could potentially explain the missing nitrogen, exists in Earth's deep mantle. Laboratory experiments on slab sediment, AOC and serpentinized mantle peridotite should be conducted systematically as a function of P–T–pH–*f*O_2_. This will enable the stability and proportion of K-bearing minerals, as well as the speciation and partitioning of nitrogen between fluids, silicate melts and K-bearing minerals, to be quantified. This will also allow the recycling efficiencies of slab nitrogen for subduction zones of different geotherms to be quantified. This will eventually assist in determining the quantity of nitrogen introduced from the Earth's surface to the mantle over geological time and also assist in investigating whether the mantle plume nitrogen is of primordial origin or of secondary origin via slab subduction. It is imperative to obtain more precise estimates of global nitrogen outflux through volcanism in order to quantify the net nitrogen ingassing of Earth's mantle. In order to gain a more comprehensive understanding of the long-term evolution of Earth's atmosphere and the exchange of nitrogen between the Earth's surface and mantle, it is necessary to obtain additional constraints on the partial pressure of N_2_ in the Earth's atmosphere at different times. Furthermore, future experiments should be conducted at conditions simulating those of the Earth's deep magma ocean in order to determine the partitioning of nitrogen, carbon and other major volatiles between the Earth's core, mantle and atmosphere. Comparative partitioning of different volatiles, in conjunction with planetary formation models, would yield new insights into the origin of Earth's volatiles in general and the origin of Earth's nitrogen in particular.

## References

[1] Catling DC, Zahnle KJ. The Archean atmosphere. Sci Adv 2020; 6: eaax1420. 10.1126/sciadv.aax1420 32133393 PMC7043912

[2] Kasting JF . Earth's early atmosphere. Science 1993; 259: 920. 10.1126/science.11536547 11536547

[3] Miller SL . A production of amino acids under possible primitive earth conditions. Science 1953; 117: 528–9. 10.1126/science.117.3046.528 13056598

[4] Goldblatt C, Claire MW, Lenton TM et al. Nitrogen-enhanced greenhouse warming on early Earth. Nat Geosci 2009; 2: 891–6. 10.1038/ngeo692

[5] Sagan C, Mullen G. Earth and Mars: evolution of atmospheres and surface temperatures. Science 1972; 177: 52–6. 10.1126/science.177.4043.52 17756316

[6] Fogel ML, Steele A. Nitrogen in extraterrestrial environments: clues to the possible presence of life. Elements 2013; 9: 367–72. 10.2113/gselements.9.5.367

[7] Rayleigh FRS . Nitrogen and argon in the Earth's crust. Nature 1938; 141: 970. 10.1038/141970a0

[8] Rayleigh FRS . Nitrogen, argon and neon in the earth's crust with applications to cosmology. Proc R Soc A-Math Phys Eng Sci 1939; 170: 451–64.

[9] Stevenson FJ . On the presence of fixed ammonium in rocks. Science 1959; 130: 221–2. 10.1126/science.130.3369.221 17816142

[10] Haendel D, Mühle K, Nitzsche H-M et al. Isotopic variations of the fixed nitrogen in metamorphic rocks. Geochim Cosmochim Acta 1986; 50: 749–58. 10.1016/0016-7037(86)90351-0

[11] Bekaert DV, Turner SJ, Broadley MW et al. Subduction-driven volatile recycling: a global mass balance. Annu Rev Earth Planet Sci 2021; 49: 37–70. 10.1146/annurev-earth-071620-055024

[12] Cartigny P, Marty B. Nitrogen isotopes and mantle geodynamics: the emergence of life and the atmosphere-crust-mantle connection. Elements 2013; 9: 359–66. 10.2113/gselements.9.5.359

[13] Bebout GE, Lazzeri KE, Geiger CA. Pathways for nitrogen cycling in Earth's crust and upper mantle: a review and new results for microporous beryl and cordierite. Am Mineral 2016; 101: 7–24. 10.2138/am-2016-5363

[14] Marty B . The origins and concentrations of water, carbon, nitrogen and noble gases on Earth. Earth Planet Sci Lett 2012; 313–14: 56–66. 10.1016/j.epsl.2011.10.040

[15] Thomazo C, Papineau D. Biogeochemical cycling of nitrogen on the early Earth. Elements 2013; 9: 345–51. 10.2113/gselements.9.5.345

[16] Canfield DE, Glazer AN, Falkowski PG. The evolution and future of Earth's nitrogen cycle. Science 2010; 330: 192–6. 10.1126/science.1186120 20929768

[17] Pinti DL, Hashizume K. Early life record from nitrogen isotopes. In: Golding S, Glikson M (eds). Earliest Life on Earth: Habitats, Environments and Methods of Detection. Dordrecht: Springer, 2011, 183–205. 10.1007/978-90-481-8794-2_8

[18] Holloway JM, Dahlgren RA. Nitrogen in rock: occurrences and biogeochemical implications. Global Biogeochem Cycles 2002; 16: 1118. 10.1029/2002GB001862

[19] Zerkle AL, Mikhail S. The geobiological nitrogen cycle: from microbes to the mantle. Geobiology 2017; 15: 343–52. 10.1111/gbi.12228 28158920 PMC5412885

[20] Pinti DL . The isotopic record of Archean nitrogen and the evolution of the early Earth. Geochemistry 2001; 1: 2.

[21] Bebout GE, Fogel ML. Nitrogen-isotope compositions of metasedimentary rocks in the Catalina Schist, California: implications for metamorphic devolatilization history. Geochim Cosmochim Acta 1992; 56: 2839–49. 10.1016/0016-7037(92)90363-N

[22] Mingram B, Bräuer K. Ammonium concentration and nitrogen isotope composition in metasedimentary rocks from different tectonometamorphic units of the European Variscan Belt. Geochim Cosmochim Acta 2001; 65: 273–87. 10.1016/S0016-7037(00)00517-2

[23] Plessen B, Harlov DE, Henry D et al. Ammonium loss and nitrogen isotopic fractionation in biotite as a function of metamorphic grade in metapelites from western Maine, USA. Geochim Cosmochim Acta 2010; 74: 4759–71. 10.1016/j.gca.2010.05.021

[24] Duit W, Jansen JBH, Van Breemen A et al. Ammonium micas in metamorphic rocks as exemplified by Dome de l'Agout (France). Am J Sci 1986; 286: 702–32. 10.2475/ajs.286.9.702

[25] Palya AP, Buick IS, Bebout GE. Storage and mobility of nitrogen in the continental crust: evidence from partially melted metasedimentary rocks, Mt. Stafford, Australia. Chem Geol 2011; 281: 211–26. 10.1016/j.chemgeo.2010.12.009

[26] Halama R, Bebout GE, Bea F. Nitrogen loss and isotopic fractionation during granulite-facies metamorphism in the lower crust (Ivrea Zone, NW Italy). Chem Geol 2021; 584: 120475. 10.1016/j.chemgeo.2021.120475

[27] Hall A . Ammonium in granites and its petrogenetic significance. Earth Sci Rev 1999; 45: 145–65. 10.1016/S0012-8252(99)00006-9

[28] Boocock TJ, Stüeken EE, Bybee GM et al. Equilibrium partitioning and isotopic fractionation of nitrogen between biotite, plagioclase, and K-feldspar during magmatic differentiation. Geochim Cosmochim Acta 2023; 356: 116–28. 10.1016/j.gca.2023.07.010

[29] Bebout GE, Fogel ML, Cartigny P. Nitrogen: highly volatile yet surprisingly compatible. Elements 2013; 9: 333–8. 10.2113/gselements.9.5.333

[30] Li K, Li L. Nitrogen enrichments in sheeted dikes and gabbros from DSDP/ODP/IODP hole 504B and 1256D: insights into nitrogen recycling in Central America and global subduction zones. Geochim Cosmochim Acta 2022; 335: 197–210. 10.1016/j.gca.2022.08.036

[31] Li K, Li L. Nitrogen enrichment in the altered upper oceanic crust: a new perspective on constraining the global subducting nitrogen budget and implications for subduction-zone nitrogen recycling. Earth Planet Sci Lett 2023; 602: 117960. 10.1016/j.epsl.2022.117960

[32] Li K, Li L. Alteration enrichment of nitrogen in the gabbroic oceanic crust: implications for global subducting nitrogen budget and subduction-zone nitrogen recycling. Geochim Cosmochim Acta 2023; 351: 96–107. 10.1016/j.gca.2023.04.029

[33] Sano Y, Takahata N, Nishio Y et al. Nitrogen recycling in subduction zones. Geophys Res Lett 1998; 25: 2289–92. 10.1029/98GL01687

[34] Sano Y, Takahata N, Nishio Y et al. Volcanic flux of nitrogen from the Earth. Chem Geol 2001; 171: 263–71. 10.1016/S0009-2541(00)00252-7

[35] Marty B . Nitrogen content of the mantle inferred from N_2_–Ar correlation in oceanic basalts. Nature 1995; 377: 326–9. 10.1038/377326a0

[36] Marty B, Humbert F. Nitrogen and argon isotopes in oceanic basalts. Earth Planet Sci Lett 1997; 152: 101–12. 10.1016/S0012-821X(97)00153-2

[37] Dauphas N, Marty B. Heavy nitrogen in carbonatites of the Kola Peninsula: a possible signature of the deep mantle. Science 1999; 286: 2488–90. 10.1126/science.286.5449.2488 10617459

[38] Cartigny P, Jendrzejewski N, Pineau F et al. Volatile (C, N, Ar) variability in MORB and the respective roles of mantle source heterogeneity and degassing: the case of the Southwest Indian Ridge. Earth Planet Sci Lett 2001; 194: 241–57. 10.1016/S0012-821X(01)00540-4

[39] Boocock TJ, Mikhail S, Boyce AJ et al. A primary magmatic source of nitrogen to Earth's crust. Nat Geosci 2023; 16: 521–6. 10.1038/s41561-023-01194-3

[40] Marty B, Zimmermann L. Volatiles (He, C, N, Ar) in mid-ocean ridge basalts: assessment of shallow-level fractionation and characterization of source composition. Geochim Cosmochim Acta 1999; 63: 3619–33. 10.1016/S0016-7037(99)00169-6

[41] Hilton DR, Fischer TP, Marty B. Noble gases and volatile recycling at subduction zones. Rev Mineral Geochem 2002; 47: 319–70. 10.2138/rmg.2002.47.9

[42] Fischer TP, Marty B. Volatile abundances in the sub-arc mantle: insights from volcanic and hydrothermal gas discharges. J Volcanol Geoth Res 2005; 140: 205–16. 10.1016/j.jvolgeores.2004.07.022

[43] Yokochi R, Marty B, Chazot G et al. Nitrogen in peridotite xenoliths: lithophile behavior and magmatic isotope fractionation. Geochim Cosmochim Acta 2009; 73: 4843–61. 10.1016/j.gca.2009.05.054

[44] Cartigny P, Farquhar J, Thomassot E et al. A mantle origin for Paleoarchean peridotitic diamonds from the Panda kimberlite. Slave Craton: evidence from 13C-, 15N- and 33,34S-stable isotope systematics. Lithos 2009; 112: 852–64. 10.1016/j.lithos.2009.06.007

[45] Cartigny P, Harris JW, Javoy M. Diamond genesis, mantle fractionations and mantle nitrogen content: a study of δ^13^ C–N concentrations in diamonds. Earth Planet Sci Lett 2001; 185: 85–98. 10.1016/S0012-821X(00)00357-5

[46] Bebout GE . Boron, lithium, and nitrogen cycling through subduction zones. Geochim Cosmochim Acta 2006; 70: A43. 10.1016/j.gca.2006.06.195

[47] Halama R, Bebout GE, John T et al. Nitrogen recycling in subducted oceanic lithosphere: the record in high-and ultrahigh-pressure metabasaltic rocks. Geochim Cosmochim Acta 2010; 74: 1636–52. 10.1016/j.gca.2009.12.003

[48] Mallik A, Rebaza AM, Kapp P et al. Metabasic rocks as important nitrogen carriers to forearc depths: implications for deep nitrogen cycling. Geochim Cosmochim Acta 2023; 361: 265–75. 10.1016/j.gca.2023.10.007

[49] Busigny V, Cartigny P, Philippot P et al. Massive recycling of nitrogen and other fluid-mobile elements (K, Rb, Cs, H) in a cold slab environment: evidence from HP to UHP oceanic metasediments of the Schistes Lustrés nappe (western Alps, Europe). Earth Planet Sci Lett 2003; 215: 27–42. 10.1016/S0012-821X(03)00453-9

[50] Bebout GE . The impact of subduction-zone metamorphism on mantle-ocean chemical cycling. Chem Geol 1995; 126: 191–218.

[51] Busigny V, Cartigny P, Philippot P. Nitrogen isotopes in ophiolitic metagabbros: a re-evaluation of modern nitrogen fluxes in subduction zones and implication for the early Earth atmosphere. Geochim Cosmochim Acta 2011; 75: 7502–21. 10.1016/j.gca.2011.09.049

[52] Li L, Li K, Li Y et al. Recommendations for offline combustion-based nitrogen isotopic analysis of silicate minerals and rocks. Rapid Commun Mass Spectrom 2021; 35: e9075. 10.1002/rcm.9075 33648023

[53] Takahata N, Nishio Y, Yoshida N et al. Precise isotopic measurements of nitrogen at the sub-nanomole level. Anal Sci 1998; 14: 485–91.

[54] Humbert F, Libourel G, France‐Lanord C et al. CO_2_-laser extraction-static mass spectrometry analysis of ultra-low concentrations of nitrogen in silicates. Geostandards Newsletter 2000; 24: 255–60. 10.1111/j.1751-908X.2000.tb00777.x

[55] Boocock TJ, Mikhail S, Prytulak J et al. Nitrogen mass fraction and stable isotope ratios for fourteen geological reference materials: evaluating the applicability of elemental analyser versus sealed tube combustion methods. Geostand Geoanal Res 2020; 44: 537–51. 10.1111/ggr.12345

[56] Barry PH, Hilton DR, Halldórsson SA et al. High precision nitrogen isotope measurements in oceanic basalts using a static triple collection noble gas mass spectrometer. Geochem Geophys Geosyst 2012; 13: Q01019. 10.1029/2011GC003878

[57] Bebout GE, Idleman BD, Li L et al. Isotope-ratio-monitoring gas chromatography methods for high-precision isotopic analysis of nanomole quantities of silicate nitrogen. Chem Geol 2007; 240: 1–10. 10.1016/j.chemgeo.2007.01.006

[58] Johnson B, Goldblatt C. The nitrogen budget of Earth. Earth Sci Rev 2015; 148: 150–73. 10.1016/j.earscirev.2015.05.006

[59] Marty B, Dauphas N. The nitrogen record of crust–mantle interaction and mantle convection from Archean to present. Earth Planet Sci Lett 2003; 206: 397–410. 10.1016/S0012-821X(02)01108-1

[60] Li K, Yu AJ, Barry PH et al. Oceanic serpentinites: a potentially critical reservoir for deep nitrogen recycling. Geology 2023; 51: 1096–100.

[61] Mallik A, Li Y, Wiedenbeck M. Nitrogen evolution within the Earth's atmosphere-mantle system assessed by recycling in subduction zones. Earth Planet Sci Lett 2018; 482: 556–66. 10.1016/j.epsl.2017.11.045

[62] Li Y, Huang R, Wiedenbeck M et al. Nitrogen distribution between aqueous fluids and silicate melts. Earth Planet Sci Lett 2015; 411: 218–28. 10.1016/j.epsl.2014.11.050

[63] Mosenfelder JL, Von Der Handt A, Füri E et al. Nitrogen incorporation in silicates and metals: results from SIMS, EPMA, FTIR, and laser-extraction mass spectrometry. Am Mineral 2019; 104: 31–46. 10.2138/am-2019-6533

[64] Gao Z, Yang Y-N, Yang S-Y et al. Experimental determination of N_2_ solubility in silicate melts and implications for N_2_–Ar–CO_2_ fractionation in magmas. Geochim Cosmochim Acta 2022; 326: 17–40. 10.1016/j.gca.2022.04.001

[65] Dalou C, Hirschmann MM, Jacobsen SD et al. Raman spectroscopy study of C-O-H-N speciation in reduced basaltic glasses: implications for reduced planetary mantles. Geochim Cosmochim Acta 2019; 265: 32–47. 10.1016/j.gca.2019.08.029

[66] Füri E, Deloule E, Dalou C. Nitrogen abundance and isotope analysis of silicate glasses by secondary ionization mass spectrometry. Chem Geol 2018; 493: 327–37. 10.1016/j.chemgeo.2018.06.008

[67] Boulliung J, Füri E, Dalou C et al. Oxygen fugacity and melt composition controls on nitrogen solubility in silicate melts. Geochim Cosmochim Acta 2020; 284: 120–33. 10.1016/j.gca.2020.06.020

[68] Li Y, Wiedenbeck M, Shcheka S et al. Nitrogen solubility in upper mantle minerals. Earth Planet Sci Lett 2013; 377: 311–23. 10.1016/j.epsl.2013.07.013

[69] Fukuyama K, Kagi H, Inoue T et al. High nitrogen solubility in stishovite (SiO_2_) under lower mantle conditions. Sci Rep 2020; 10: 10897. 10.1038/s41598-020-67621-2 32616729 PMC7331719

[70] Yoshioka T, Wiedenbeck M, Shcheka S et al. Nitrogen solubility in the deep mantle and the origin of Earth's primordial nitrogen budget. Earth Planet Sci Lett 2018; 488: 134–43. 10.1016/j.epsl.2018.02.021

[71] Watenphul A, Wunder B, Wirth R et al. Ammonium-bearing clinopyroxene: a potential nitrogen reservoir in the Earth's mantle. Chem Geol 2010; 270: 240–8. 10.1016/j.chemgeo.2009.12.003

[72] Libourel G, Marty B, Humbert F. Nitrogen solubility in basaltic melt. Part I. Effect of oxygen fugacity. Geochim Cosmochim Acta 2003; 67: 4123–35. 10.1016/S0016-7037(03)00259-X

[73] Bernadou F, Gaillard F, Füri E et al. Nitrogen solubility in basaltic silicate melt—implications for degassing processes. Chem Geol 2021; 573: 120192. 10.1016/j.chemgeo.2021.120192

[74] Fukuyama K, Kagi H, Inoue T et al. Temperature dependence of nitrogen solubility in bridgmanite and evolution of nitrogen storage capacity in the lower mantle. Sci Rep 2023; 13: 3537. 10.1038/s41598-023-30556-5 36864194 PMC9981615

[75] Sokol AG, Kupriyanov IN, Kotsuba DA et al. Nitrogen storage capacity of phengitic muscovite and K-cymrite under the conditions of hot subduction and ultra high pressure metamorphism. Geochim Cosmochim Acta 2023; 355: 89–109. 10.1016/j.gca.2023.06.026

[76] Jackson CRM, Cottrell E. Nitrogen partitioning between silicate phases and aqueous fluid depends on concentration. Geochim Cosmochim Acta 2023; 354: 1–12. 10.1016/j.gca.2023.05.017

[77] Förster MW, Foley SF, Alard O et al. Partitioning of nitrogen during melting and recycling in subduction zones and the evolution of atmospheric nitrogen. Chem Geol 2019; 525: 334–42. 10.1016/j.chemgeo.2019.07.042

[78] Shi L, Lu W, Kagoshima T et al. Nitrogen isotope evidence for Earth's heterogeneous accretion of volatiles. Nat Commun 2022; 13: 4769. 10.1038/s41467-022-32516-5 35970934 PMC9378614

[79] Dalou C, Füri E, Deligny C et al. Redox control on nitrogen isotope fractionation during planetary core formation. Proc Natl Acad Sci USA 2019; 116: 14485–94. 10.1073/pnas.1820719116 31262822 PMC6642344

[80] Grewal DS, Dasgupta R, Holmes AK et al. The fate of nitrogen during core-mantle separation on Earth. Geochim Cosmochim Acta 2019; 251: 87–115. 10.1016/j.gca.2019.02.009 35153302 PMC8833147

[81] Grewal DS, Dasgupta R, Sun C et al. Delivery of carbon, nitrogen, and sulfur to the silicate Earth by a giant impact. Sci Adv 2019; 5: eaau3669. 10.1126/sciadv.aau3669 30746449 PMC6357864

[82] Li Y, Marty B, Shcheka S et al. Nitrogen isotope fractionation during terrestrial core-mantle separation. Geochem Perspect Lett 2016; 2: 138–47. 10.7185/geochemlet.1614

[83] Li Y, Wiedenbeck M, Monteleone B et al. Nitrogen and carbon fractionation in planetary magma oceans and origin of the superchondritic C/N ratio in the bulk silicate Earth. Earth Planet Sci Lett 2023; 605: 118032. 10.1016/j.epsl.2023.118032

[84] Grewal DS, Dasgupta R, Hough T et al. Rates of protoplanetary accretion and differentiation set nitrogen budget of rocky planets. Nat Geosci 2021; 14: 369–76. 10.1038/s41561-021-00733-0 34163536 PMC8216213

[85] Speelmanns IM, Schmidt MW, Liebske C. The almost lithophile character of nitrogen during core formation. Earth Planet Sci Lett 2019; 510: 186–97. 10.1016/j.epsl.2019.01.004

[86] Jackson CRM, Cottrell E, Du Z et al. High pressure redistribution of nitrogen and sulfur during planetary stratification. Geochem Perspect Lett 2021; 18: 37–42.

[87] Rustioni G, Wiedenbeck M, Miyajima N et al. Magnesiowüstite as a major nitrogen reservoir in Earth's lowermost mantle. Geochem Perspect Lett 2024; 28: 43–7. 10.7185/geochemlet.2401

[88] Ussiri D, Lal R. Global Nitrogen Cycle. In: Ussiri D, and Lal R (eds). Soil Emission of Nitrous Oxide and its Mitigation. Netherlands: Springer, 2013, 29–62.

[89] Rudnick RL, Gao S. Composition of the continental crust. Treatise on Geochemistry 2014; 4: 1–51. 10.1016/B978-0-08-095975-7.00301-6

[90] Labidi J, Barry PH, Bekaert DV et al. Hydrothermal ^15^N^15^N abundances constrain the origins of mantle nitrogen. Nature 2020; 580: 367–71. 10.1038/s41586-020-2173-4 32296193

[91] Halliday AN . The origins of volatiles in the terrestrial planets. Geochim Cosmochim Acta 2013; 105: 146–71. 10.1016/j.gca.2012.11.015

[92] Hirschmann MM . Comparative deep Earth volatile cycles: the case for C recycling from exosphere/mantle fractionation of major (H_2_O, C, N) volatiles and from H_2_O/Ce, CO_2_/Ba, and CO_2_/Nb exosphere ratios. Earth Planet Sci Lett 2018; 502: 262–73. 10.1016/j.epsl.2018.08.023

[93] Grewal DS, Dasgupta R, Marty B. A very early origin of isotopically distinct nitrogen in inner Solar System protoplanets. Nat Astron 2021; 5: 356–64. 10.1038/s41550-020-01283-y

[94] Andersen T, Burke EAJ, Neumann E-R. Nitrogen-rich fluid in the upper mantle: fluid inclusions in spinel dunite from Lanzarote, Canary Islands. Contrib Mineral Petrol 1995; 120: 20–8. 10.1007/BF00311005

[95] Bebout GE, Ryan JG, Leeman WP et al. Fractionation of trace elements by subduction-zone metamorphism—Effect of convergent-margin thermal evolution. Earth Planet Sci Lett 1999; 171: 63–81. 10.1016/S0012-821X(99)00135-1

[96] Liu W, Fei P. Methane-rich fluid inclusions from ophiolitic dunite and post-collisional mafic–ultramafic intrusion: the mantle dynamics underneath the Palaeo-Asian Ocean through to the post-collisional period. Earth Planet Sci Lett 2006; 242: 286–301. 10.1016/j.epsl.2005.11.059

[97] Nishizawa M, Sano Y, Ueno Y et al. Speciation and isotope ratios of nitrogen in fluid inclusions from seafloor hydrothermal deposits at ∼ 3.5 Ga. Earth Planet Sci Lett 2007; 254: 332–44. 10.1016/j.epsl.2006.11.044

[98] Berkesi M, Káldos R, Park M et al. Detection of small amounts of N_2_ in CO_2_-rich high-density fluid inclusions from mantle xenoliths. Eur J Mineral 2017; 29: 423–31. 10.1127/ejm/2017/0029-2615

[99] Fischer TP . Fluxes of volatiles (H_2_O, CO_2_, N_2_, Cl, F) from arc volcanoes. Geochem J 2008; 42: 21–38. 10.2343/geochemj.42.21

[100] Mather TA, Allen AG, Davison BM et al. Nitric acid from volcanoes. Earth Planet Sci Lett 2004; 218: 17–30.

[101] Kaminsky F, Wirth R. Nitrides and carbonitrides from the lowermost mantle and their importance in the search for Earth's “lost” nitrogen. Am Mineral 2017; 102: 1667–76. 10.2138/am-2017-6101

[102] Adler JF, Williams Q. A high-pressure X-ray diffraction study of iron nitrides: implications for Earth's core. J Geophys Res 2005; 110.: B01203. 10.1029/2004JB003103

[103] Daviau K, Fischer RA, Brennan MC et al. Equation of State of TiN at high pressures and temperatures: a possible host for nitrogen in planetary mantles. J Geophys Res-Solid Earth 2021; 126: e2020JB020074. 10.1029/2020JB020074

[104] Minobe S, Nakajima Y, Hirose K et al. Stability and compressibility of a new iron-nitride β-Fe_7_N_3_ to core pressures. Geophys Res Lett 2015; 42: 5206–11. 10.1002/2015GL064496

[105] Pangritz P, Rohrbach A, Vollmer C et al. The Fe(Ni)–C–N-phase diagram at 10 GPa—Implications for nitrogen and carbon storage in the deep mantle. Contrib Mineral Petrol 2023; 179: 3.

[106] Siwka J, Hutny A. An universal formula for the calculation of nitrogen solubility in liquid nitrogen-alloyed steels. Metalurgija 2009; 48: 23–7.

[107] Roskosz M, Bouhifd MA, Jephcoat AP et al. Nitrogen solubility in molten metal and silicate at high pressure and temperature. Geochim Cosmochim Acta 2013; 121: 15–28. 10.1016/j.gca.2013.07.007

[108] Speelmanns IM, Schmidt MW, Liebske C. Nitrogen solubility in core materials. Geophys Res Lett 2018; 45: 7434–43. 10.1029/2018GL079130

[109] Sokol AG, Khokhryakov AF, Borzdov YM et al. Solubility of carbon and nitrogen in a sulfur-bearing iron melt: constraints for siderophile behavior at upper mantle conditions. Am Mineral 2019; 104: 1857–65. 10.2138/am-2019-7103

[110] Labidi J . The origin of nitrogen in Earth's mantle: constraints from basalts ^15^N/^14^N and N_2_/^3^He ratios. Chem Geol 2022; 597: 120780. 10.1016/j.chemgeo.2022.120780

[111] Liu W, Feng L, Liu X et al. Nitrogen and oxygen isotopic profiles and redox structure across the Chinese Altay reveal the source rocks of granites, core of the Central Asian Orogenic Belt. Lithos 2023; 462–3: 107392. 10.1016/j.lithos.2023.107392

[112] Halama R, Bebout GE, John T et al. Nitrogen recycling in subducted mantle rocks and implications for the global nitrogen cycle. Int J Earth Sci 2014; 103: 2081–99. 10.1007/s00531-012-0782-3

[113] Li K, Li G-Y, Du Y-F et al. Intraslab remobilization of nitrogen during early subduction facilitates deep nitrogen recycling: insights from the blueschists in the Heilongjiang Complex in NE China. Chem Geol 2021; 583: 120474. 10.1016/j.chemgeo.2021.120474

[114] Li L, Zheng Y-F, Cartigny P et al. Anomalous nitrogen isotopes in ultrahigh-pressure metamorphic rocks from the Sulu orogenic belt: effect of abiotic nitrogen reduction during fluid–rock interaction. Earth Planet Sci Lett 2014; 403: 67–78. 10.1016/j.epsl.2014.06.029

[115] Palot M, Cartigny P, Harris JW et al. Evidence for deep mantle convection and primordial heterogeneity from nitrogen and carbon stable isotopes in diamond. Earth Planet Sci Lett 2012; 357–8: 179–93. 10.1016/j.epsl.2012.09.015

[116] Stachel T, Cartigny P, Chacko T et al. Carbon and nitrogen in mantle-derived diamonds. Rev Mineral Geochem 2022; 88: 809–75. 10.2138/rmg.2022.88.15

[117] Javoy M . The major volatile elements of the Earth: their origin, behavior, and fate. Geophys Res Lett 1997; 24: 177–80. 10.1029/96GL03931

[118] Grady MM, Wright IP, Carr LP et al. Compositional differences in enstatite chondrites based on carbon and nitrogen stable isotope measurements. Geochim Cosmochim Acta 1986; 50: 2799–813. 10.1016/0016-7037(86)90228-0

[119] Kerridge JF . Carbon, hydrogen and nitrogen in carbonaceous chondrites: abundances and isotopic compositions in bulk samples. Geochim Cosmochim Acta 1985; 49: 1707–14. 10.1016/0016-7037(85)90141-3 11539652

[120] Cartigny P, Ader M. A comment on “the nitrogen record of crust-mantle interaction and mantle convection from Archean to present” by B. Marty and N. Dauphas Earth Planet. Sci. Lett. 206(2003) 397–410—discussion. Earth Planet Sci Lett 2003; 216: 425–32. 10.1016/S0012-821X(03)00505-3

[121] Dauphas N . The isotopic nature of the Earth's accreting material through time. Nature 2017; 541: 521–4. 10.1038/nature20830 28128239

[122] Fischer-Gödde M, Kleine T. Ruthenium isotopic evidence for an inner Solar System origin of the late veneer. Nature 2017; 541: 525–7. 10.1038/nature21045 28128236

[123] Tolstikhin IN, Marty B. The evolution of terrestrial volatiles: a view from helium, neon, argon and nitrogen isotope modelling. Chem Geol 1998; 147: 27–52. 10.1016/S0009-2541(97)00170-8

[124] Li Y, Marty B, Shcheka S et al. Nitrogen isotope fractionation during terrestrial core-mantle separation. Geochem Perspect Lett 2016; 2: 138–47. 10.7185/geochemlet.1614

[125] Kerrich R, Jia Y. A comment on “The nitrogen record of crust–mantle interaction and mantle convection from Archean to present” by B. Marty and N. Dauphas [Earth Planet. Sci. Lett. 206 (2003) 397–410]. Earth Planet Sci Lett 2004; 225: 435–40. 10.1016/j.epsl.2004.07.004

[126] Walsh KJ, Morbidelli A, Raymond SN et al. A low mass for Mars from Jupiter's early gas-driven migration. Nature 2011; 475: 206. 10.1038/nature10201 21642961

[127] Morbidelli A, Chambers J, Lunine JI et al. Source regions and timescales for the delivery of water to the Earth. Meteorit Planet Sci 2000; 35: 1309–20. 10.1111/j.1945-5100.2000.tb01518.x

[128] Fujiya W, Hoppe P, Ushikubo T et al. Migration of D-type asteroids from the outer Solar System inferred from carbonate in meteorites. Nat Astron 2019; 3: 910–5.

[129] Alexander CMO'D. The origin of inner Solar System water. Philos Trans A Math Phys Eng Sci 2017; 375: 20150384. 10.1098/rsta.2015.0384 PMC5394251 28416723

[130] Hirschmann MM . Constraints on the early delivery and fractionation of Earth's major volatiles from C/H, C/N, and C/S ratios. Am Mineral 2016; 101: 540–53. 10.2138/am-2016-5452

[131] Bergin EA, Blake GA, Ciesla F et al. Tracing the ingredients for a habitable earth from interstellar space through planet formation. Proc Natl Acad Sci USA 2015; 112: 8965–70. 10.1073/pnas.1500954112 26150527 PMC4517224

[132] Albarède F . Volatile accretion history of the terrestrial planets and dynamic implications. Nature 2009; 461: 1227–33. 10.1038/nature08477 19865163

[133] Braukmüller N, Wombacher F, Funk C et al. Earth's volatile element depletion pattern inherited from a carbonaceous chondrite-like source. Nat Geosci 2019; 12: 564–8. 31249609 10.1038/s41561-019-0375-x PMC6597353

[134] Wang Z, Becker H. Ratios of S, Se and Te in the silicate Earth require a volatile-rich late veneer. Nature 2013; 499: 328–31. 10.1038/nature12285 23868263

[135] Schönbächler M, Carlson RW, Horan MF et al. Heterogeneous accretion and the moderately volatile element budget of Earth. Science 2010; 328: 884–7. 10.1126/science.1186239 20466929

[136] Rubie DC, Jacobson SA, Morbidelli A et al. Accretion and differentiation of the terrestrial planets with implications for the compositions of early-formed Solar System bodies and accretion of water. Icarus 2015; 248: 89–108. 10.1016/j.icarus.2014.10.015

[137] Sakuraba H, Kurokawa H, Genda H et al. Numerous chondritic impactors and oxidized magma ocean set Earth's volatile depletion. Sci Rep 2021; 11: 20894. 10.1038/s41598-021-99240-w 34686749 PMC8536732

[138] Li Y, Dasgupta R, Tsuno K et al. Carbon and sulfur budget of the silicate Earth explained by accretion of differentiated planetary embryos. Nat Geosci 2016; 9: 781–5. 10.1038/ngeo2801

[139] Budde G, Burkhardt C, Kleine T. Molybdenum isotopic evidence for the late accretion of outer Solar System material to Earth. Nat Astron 2019; 3: 736–41. 10.1038/s41550-019-0779-y

[140] Marty B, Chaussidon M, Wiens RC et al. A 15N-poor isotopic composition for the solar system as shown by Genesis solar wind samples. Science 2011; 332: 1533–6. 10.1126/science.1204656 21700869

[141] Füri E, Marty B. Nitrogen isotope variations in the Solar System. Nat Geosci 2015; 8: 515–22. 10.1038/ngeo2451

[142] Marty B, Avice G, Sano Y et al. Origins of volatile elements (H, C, N, noble gases) on Earth and Mars in light of recent results from the ROSETTA cometary mission. Earth Planet Sci Lett 2016; 441: 91–102. 10.1016/j.epsl.2016.02.031

[143] Broadley MW, Bekaert DV, Piani L et al. Origin of life-forming volatile elements in the inner Solar System. Nature 2022; 611: 245–55. 10.1038/s41586-022-05276-x 36352134

[144] Piani L, Marrocchi Y, Rigaudier T et al. Earth's water may have been inherited from material similar to enstatite chondrite meteorites. Science 2020; 369: 1110–13. 10.1126/science.aba1948 32855337

[145] Elkins-Tanton LT . Magma oceans in the inner Solar system. Annu Rev Earth Planet Sci 2012; 40: 113–39. 10.1146/annurev-earth-042711-105503

[146] Gu JT, Peng B, Ji X et al. Composition of Earth's initial atmosphere and fate of accreted volatiles set by core formation and magma ocean redox evolution. Earth Planet Sci Lett 2024; 629: 118618. 10.1016/j.epsl.2024.118618

[147] Chen H, Jacobson SA. Impact induced atmosphere-mantle exchange sets the volatile elemental ratios on primitive Earths. Earth Planet Sci Lett 2022; 594: 117741. 10.1016/j.epsl.2022.117741

[148] Li Y, Dasgupta R, Tsuno K. The effects of sulfur, silicon, water, and oxygen fugacity on carbon solubility and partitioning in Fe-rich alloy and silicate melt systems at 3 GPa and 1600°C: implications for core–mantle differentiation and degassing of magma oceans and reduced planetary mantles. Earth Planet Sci Lett 2015; 415: 54–66.

[149] Dalou C, Hirschmann MM, von der Handt A et al. Nitrogen and carbon fractionation during core–mantle differentiation at shallow depth. Earth Planet Sci Lett 2017; 458: 141–51.

[150] Dasgupta R, Falksen E, Pal A et al. The fate of nitrogen during parent body partial melting and accretion of the inner solar system bodies at reducing conditions. Geochim Cosmochim Acta 2022; 336: 291–307. 10.1016/j.gca.2022.09.012

[151] Grewal DS, Sun T, Aithala S et al. Limited nitrogen isotopic fractionation during core-mantle differentiation in rocky protoplanets and planets. Geochim Cosmochim Acta 2022; 338: 347–64. 10.1016/j.gca.2022.10.025

[152] Rubie DC, Frost DJ, Mann U et al. Heterogeneous accretion, composition and core–mantle differentiation of the Earth. Earth Planet Sci Lett 2011; 301: 31–42. 10.1016/j.epsl.2010.11.030

[153] Pearson VK, Sephton MA, Franchi IA et al. Carbon and nitrogen in carbonaceous chondrites: elemental abundances and stable isotopic compositions. Meteorit Planet Sci 2006; 41: 1899–918. 10.1111/j.1945-5100.2006.tb00459.x

[154] Miyazaki A, Hiyagon H, Sugiura N et al. Solubilities of nitrogen and noble gases in silicate melts under various oxygen fugacities: implications for the origin and degassing history of nitrogen and noble gases in the Earth. Geochim Cosmochim Acta 2004; 68: 387–401. 10.1016/S0016-7037(03)00484-8

[155] Roskosz M, Mysen BO, Cody GD. Dual speciation of nitrogen in silicate melts at high pressure and temperature: an experimental study. Geochim Cosmochim Acta 2006; 70: 2902–18. 10.1016/j.gca.2006.03.001

[156] Mysen BO, Yamashita S, Chertkova N. Solubility and solution mechanisms of NOH volatiles in silicate melts at high pressure and temperature-amine groups and hydrogen fugacity. Am Mineral 2008; 93: 1760–70. 10.2138/am.2008.2879

[157] Mysen BO, Fogel ML. Nitrogen and hydrogen isotope compositions and solubility in silicate melts in equilibrium with reduced (N+H)-bearing fluids at high pressure and temperature: effects of melt structure. Am Mineral 2010; 95: 987–99. 10.2138/am.2010.3364

[158] Kadik AA, Kurovskaya NA, Ignat'ev YA et al. Influence of oxygen fugacity on the solubility of nitrogen, carbon, and hydrogen in FeO-Na_2_O-SiO_2_-Al_2_O_3_ melts in equilibrium with metallic iron at 1.5 GPa and 1400°C. Geochem Int 2011; 49: 429–38. 10.1134/S001670291105003X

[159] Kadik AA, Koltashev VV, Kryukova EB et al. Solubility of nitrogen, carbon, and hydrogen in FeO–Na_2_O–Al_2_O_3_–SiO_2_ melt and liquid iron alloy: influence of oxygen fugacity. Geochem Int 2015; 53: 849–68. 10.1134/S001670291510002X

[160] Keppler H, Cialdella L, Couffignal F et al. The solubility of N_2_ in silicate melts and nitrogen partitioning between upper mantle minerals and basalt. Contrib Mineral Petrol 2022; 177: 83. 10.1007/s00410-022-01948-z

[161] Grewal DS, Dasgupta R, Farnell A. The speciation of carbon, nitrogen, and water in magma oceans and its effect on volatile partitioning between major reservoirs of the Solar System rocky bodies (vol 280, pg 281, 2020). Geochim Cosmochim Acta 2020; 285: 274. 10.1016/j.gca.2020.06.031

[162] Frost DJ, Mccammon CA. The redox state of Earth's mantle. Annu Rev Earth Planet Sci 2008; 36: 389–420. 10.1146/annurev.earth.36.031207.124322

[163] Halama R, Bebout GE, Marschall HR et al. Fluid-induced breakdown of white mica controls nitrogen transfer during fluid–rock interaction in subduction zones. Int Geol Rev 2017; 59: 702–20. 10.1080/00206814.2016.1233834

[164] Harris BJR, De Hoog JCM, Halama R. The behaviour of nitrogen during subduction of oceanic crust: insights from in situ SIMS analyses of high-pressure rocks. Geochim Cosmochim Acta 2022; 321: 16–34. 10.1016/j.gca.2022.01.018

[165] Liu W, Yang Y, Busigny V et al. Intimate link between ammonium loss of phengite and the deep Earth's water cycle. Earth Planet Sci Lett 2019; 513: 95–102. 10.1016/j.epsl.2019.02.022

[166] Poli S, Schmidt MW. Petrology of subducted slabs. Annu Rev Earth Planet Sci 2002; 30: 207–35. 10.1146/annurev.earth.30.091201.140550

[167] Domanik KJ, Holloway JR. The stability and composition of phengitic muscovite and associated phases from 5.5 to 11 GPa: implications for deeply subducted sediments. Geochim Cosmochim Acta 1996; 60: 4133–50. 10.1016/S0016-7037(96)00241-4

[168] Schmidt MW . Experimental constraints on recycling of potassium from subducted oceanic crust. Science 1996; 272: 1927. 10.1126/science.272.5270.1927 8662494

[169] Mitchell EC, Fischer TP, Hilton DR et al. Nitrogen sources and recycling at subduction zones: insights from the Izu-Bonin-Mariana arc. Geochem Geophys Geosyst 2010; 11: Q02X11. 10.1029/2009GC002783

[170] Elkins LJ, Fischer TP, Hilton DR et al. Tracing nitrogen in volcanic and geothermal volatiles from the Nicaraguan volcanic front. Geochim Cosmochim Acta 2006; 70: 5215–35. 10.1016/j.gca.2006.07.024

[171] Fischer TP, Hilton DR, Zimmer MM et al. Subduction and recycling of nitrogen along the Central American margin. Science 2002; 297: 1154–7. 10.1126/science.1073995 12183622

[172] Jia Y, Kerrich R, Gupta AK et al. 15N-enriched Gondwana lamproites, eastern India: crustal N in the mantle source. Earth Planet Sci Lett 2003; 215: 43–56. 10.1016/S0012-821X(03)00426-6

[173] Cartigny P, Chinn I, Viljoen KS(F) et al. Early proterozoic ultrahigh pressure metamorphism: evidence from microdiamonds. Science 2004; 304: 853–5. 10.1126/science.1094668 15131301

[174] Smart KA, Tappe S, Stern RA et al. Early archaean tectonics and mantle redox recorded in Witwatersrand diamonds. Nat Geosci 2016; 9: 255–9. 10.1038/ngeo2628

[175] Barry PH, Hilton DR. Release of subducted sedimentary nitrogen throughout Earth's mantle. Geochem Perspect Lett 2016; 2: 148–59. 10.7185/geochemlet.1615

[176] Labidi J, Young ED. The origin and dynamics of nitrogen in the Earth's mantle constrained by ^15^N^15^N in hydrothermal gases. Chem Geol 2022; 591: 120709. 10.1016/j.chemgeo.2022.120709

[177] Watenphul A, Wunder B, Heinrich W. High-pressure ammonium-bearing silicates: implications for nitrogen and hydrogen storage in the Earth's mantle. Am Mineral 2009; 94: 283–92. 10.2138/am.2009.2995

[178] Li Y, Keppler H. Nitrogen speciation in mantle and crustal fluids. Geochim Cosmochim Acta 2014; 129: 13–32. 10.1016/j.gca.2013.12.031

[179] Mikhail S, Barry PH, Sverjensky DA. The relationship between mantle pH and the deep nitrogen cycle. Geochim Cosmochim Acta 2017; 209: 149–60. 10.1016/j.gca.2017.04.007

[180] Chen Q, Zhang Z, Wang Z et al. In situ Raman spectroscopic study of nitrogen speciation in aqueous fluids under pressure. Chem Geol 2019; 506: 51–7. 10.1016/j.chemgeo.2018.12.016

[181] Pöter B, Gottschalk M, Heinrich W. Experimental determination of the ammonium partitioning among muscovite, K-feldspar, and aqueous chloride solutions. Lithos 2004; 74: 67–90. 10.1016/j.lithos.2004.01.002

[182] Jackson CRM, Cottrell E, Andrews B. Warm and oxidizing slabs limit ingassing efficiency of nitrogen to the mantle. Earth Planet Sci Lett 2021; 553: 116615. 10.1016/j.epsl.2020.116615

[183] Förster MW, Chen C, Foley SF et al. Fluid loss to the fore-arc controls the recycling efficiency of nitrogen in subduction zones. Chem Geol 2024; 650: 121985. 10.1016/j.chemgeo.2024.121985

[184] Mallik A, Li Y, Wiedenbeck M. Nitrogen evolution within the Earth's atmosphere–mantle system assessed by recycling in subduction zones. Earth Planet Sci Lett 2018; 482: 556–66. 10.1016/j.epsl.2017.11.045

[185] Som SM, Catling DC, Harnmeijer JP et al. Air density 2.7 billion years ago limited to less than twice modern levels by fossil raindrop imprints. Nature 2012; 484: 359–62. 10.1038/nature10890 22456703

[186] Som SM, Buick R, Hagadorn JW et al. Earth's air pressure 2.7 billion years ago constrained to less than half of modern levels. Nat Geosci 2016; 9: 448–51. 10.1038/ngeo2713

[187] Marty B, Zimmermann L, Pujol M et al. Nitrogen isotopic composition and density of the Archean atmosphere. Science 2013; 342: 101–4. 10.1126/science.1240971 24051244

[188] Sano Y, Pillinger CT. Nitrogen isotopes and N2/Ar ratios in cherts. An attempt to measure time evolution of atmospheric .DELTA.^15^N value. Geochem J 1990; 24: 315–25. 10.2343/geochemj.24.315

[189] Johnson BW, Goldblatt C. A secular increase in continental crust nitrogen during the precambrian. Geochem Perspect Lett 2017; 4: 24–8. 10.7185/geochemlet.1731

[190] Kadik AA, Litvin YA, Koltashev VV et al. Solution behavior of reduced N–H–O volatiles in FeO–Na_2_O–SiO_2_–Al_2_O_3_ melt equilibrated with molten Fe alloy at high pressure and temperature. Phys Earth Planet In 2013; 214: 14–24. 10.1016/j.pepi.2012.10.013

[191] Dalou C, Deligny C, Füri E. Nitrogen isotope fractionation during magma ocean degassing: tracing the composition of early Earth's atmosphere. Geochem Perspect Lett 2022; 20: 27–31. 10.7185/geochemlet.2204

[192] Kupriyanov IN, Sokol AG, Seryotkin YV et al. Nitrogen fractionation in mica metapelite under hot subduction conditions: implications for nitrogen ingassing to the mantle. Chem Geol 2023; 628: 121476. 10.1016/j.chemgeo.2023.121476

